# AMPK tunes reproductive gene expression and small RNA homeostasis to mediate timely germ cell development

**DOI:** 10.1093/nar/gkag555

**Published:** 2026-06-08

**Authors:** Sabih Rashid, Fabian Braukmann, Eric A Miska, Richard Roy

**Affiliations:** Department of Biology, McGill University; Montreal, H3A 1B1, Canada; Department of Biochemistry, University of Cambridge, Cambridge, CB2 1GA, United Kingdom; The Gurdon Institute, University of Cambridge, Cambridge, CB2 1QN, United Kingdom; Department of Biochemistry, University of Cambridge, Cambridge, CB2 1GA, United Kingdom; The Gurdon Institute, University of Cambridge, Cambridge, CB2 1QN, United Kingdom; Department of Biology, McGill University; Montreal, H3A 1B1, Canada; Stewart Biology Building, 1205 Docteur Penfield, Montréal, Quebec H3A 1B1, Canada

## Abstract

Developmental plasticity allows organisms to adapt to environmental stressors and improve fitness. *Caenorhabditis elegans* can enter an alternative larval stage, the dauer, marked by developmental quiescence and complete transcriptomic remodeling. The energy modulator AMPK is required for animals to passage through dauer while preserving germline quiescence. Without AMPK, dauer animals exhibit reproductive defects, which are partially suppressed by disabling small RNA pathway components. To understand how the loss of AMPK affects RNA homeostasis and gene expression, we performed transcriptomic analysis of AMPK mutants. We reveal that over 60% of the *C. elegans* genes are affected by the loss of AMPK during the dauer stage. This is accompanied by a widespread increase in reproductive gene expression in dauer, as well as the upregulation of most small RNAs and small RNA pathway components, particularly the spermatogenic Argonautes ALG-3/4. Eliminating ALG-3/4 corrects several reproductive defects, including premature dauer spermatogenesis, and partially restores fertility. Notably, decreasing global small RNA biogenesis also partially corrects these phenotypes, possibly by destabilizing their associated Argonaute effectors. Our work highlights a role for AMPK in the temporal regulation of small RNA populations to establish a delicate balance that is essential for reproductive fitness following quiescence during periods of stress.

## Graphical Abstract

**Graphical Abstract:**
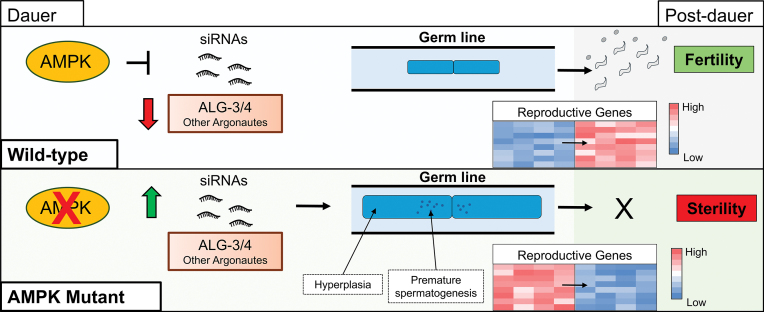

## Introduction

The proper development of organisms is closely tied to their energy and nutrient status [1–3]. Without meeting appropriate metabolic requirements, animals may exhibit slowed growth, developmental defects, and some can even enter a state of arrested development [4–6]. Reproductive development is particularly susceptible to these metabolic demands across several animal kingdoms, as many aspects of the reproductive process, from spermatogenesis to embryo production, are dependent on adequate energy and nutrient balance [7, 8]. Insufficient nutritional resources can culminate in decreased fertility, and in extreme cases can lead to defects in the immediate offspring [9, 10], or in subsequent generations [11, 12]. Therefore, organisms have evolved various means of adjusting their development and reproductive onset in response to changes in resource availability, and hence their metabolic status, in order to optimize reproductive fitness [13–16].

Despite its mosaic development and invariable lineage, development of the nematode *Caenorhabditis elegans* can nevertheless be dramatically influenced by its environment, and during these challenges it must rely on its intrinsic forms of developmental plasticity to survive and adapt to harsh growing conditions. Perhaps the best characterized of these is the alternative third larval stage called the “dauer”. This larval stage is executed when *C. elegans* are confronted with specific environmental stressors, namely a lack of energy resources, or overcrowding, and animals can remain quiescent in this stage for up to several months until conditions improve [17]. These and other adaptations allow *C. elegans* to survive for extended periods even without external nutrition, and often with little to no consequence to their reproductive viability.

The ability to mount an adaptive response to these cues requires the function of several factors, many of which confer metabolic adjustment. One such modulator is the highly conserved AMP-activated protein kinase (AMPK), which responds to a decrease in cellular energy. Through phosphorylation of specific protein targets, AMPK enhances cellular ATP production by increasing catabolism [18], all the while blocking most anabolic processes. AMPK functions in conjunction with numerous other signaling pathways involved in growth and cell cycle regulation, including TOR and insulin/IGF-1 (IIS) signaling [19, 20]. Moreover, beyond its critical role in growth, AMPK has well characterized functions in the regulation of lipid metabolism, autophagy, longevity, and reproduction. Its function is crucial for animals to adjust to changing metabolic conditions, and in numerous species the kinase has a role in regulating reproductive fitness during these challenges [21, 22]. Loss of AMPK severely reduces male fertility in humans, as the protein has been shown to be required for healthy sperm function, specifically for spermatozoa motility [23, 24]. AMPK also regulates follicular development in mammals through interactions with the mTOR pathway, although there appear to be significant differences in its role and importance in this function between species [25, 26].

In *C. elegans*, AMPK has been implicated in the regulation of lifespan and metabolism, and is required for the increased longevity seen in dietary restricted animals [21, 26–32]. Moreover, AMPK also promotes survival of animals during nutrient stress by regulating stem cell quiescence, with loss-of-function AMPK mutants exhibiting severe fertility defects when undergoing starvation [12, 28, 33]. Remarkably, unlike most other animals, *C. elegans* AMPK-deficient mutants do not exhibit any obvious reproductive defects when grown in optimal conditions, suggesting there may be compensatory mechanisms for the lack of AMPK activity, or that AMPK is not strictly required for standard reproductive development during replete conditions. However, AMPK-deficient animals exhibit severe defects during starvation or when passaged through the dauer stage, including greatly reduced fertility following their recovery from the arrest [12, 21, 28, 34]. The dauer stage requires significant remodeling at the level of gene expression, accompanied by major metabolic shifts that enhance survival during this long nonfeeding stage. This inappropriate adjustment in gene expression accounts for the broad physiological and developmental impacts that are observed during this and subsequent stages in animals that have reduced or absent AMPK function [35, 36].

AMPK mutant dauer larvae exhibit shortened survival, germline hyperplasia, premature spermatogenesis, and following their recovery from the diapause, they develop somatic defects in addition to the aforementioned sterility [34]. It is likely that these phenotypes reflect the inability of the mutant animals to adjust to the stresses associated with the dauer stage. It is unclear how the loss of AMPK signaling manifests as defects in germline quiescence during the dauer stage and subsequent post-dauer reproductive development, although prior observations suggest that these germ line-associated abnormalities may arise through the inappropriate regulation of small RNA pathway components [34].

Small noncoding RNAs mediate many aspects of *C. elegans* development and physiology [37]. The timing of development is regulated by key microRNAs (miRNAs) that are expressed in a punctuated manner to trigger stage specific events [38, 39]. Core RNA interference (RNAi) pathways, including conserved Argonautes and factors such as Dicer, are also essential for the production and activity of small RNAs which mediate developmental progression [40]. A significant portion of small RNAs within *C. elegans* are expressed in the germ cells, particularly during germline development and early embryogenesis [41–44], and disruption of many Argonautes or factors involved in small RNA biogenesis compromises fertility in animals, particularly at elevated temperatures [45, 46]. The *mutator* complex, which comprises MUT-16 and its associated proteins, is required for the function of small RNAs within the germ line [47], and is itself regulated by factors such as developmental stage and RNA populations [48]. This suggests small RNA activity is essential for organisms to respond to environmental stressors and modulate their development. Our previous findings implicated small RNA pathway function in the reproductive phenotypes seen in AMPK mutants [34]. Understanding the changes that occur in the RNA landscape in AMPK mutants could provide clues regarding the mechanisms that AMPK affects to regulate development in response to various sources of environmental stress.

Here, we analyse the global changes in gene expression in AMPK-deficient dauer and post-dauer animals to better understand the molecular genetic underpinnings of the many documented phenotypes in these mutants, and to determine how the absence of AMPK influences diverse sets of small RNA populations. Through gene enrichment analysis, we identified the major subsets of genes that are differentially expressed in AMPK-deficient animals, while validation of our findings through genetic and cell biological analyses demonstrated a clear link between most of the mutant phenotypes and the changes in gene expression induced by the loss of AMPK. In particular, we highlight the impact of small RNA pathways and their misregulation in AMPK mutants, which in turn influences many of the observed reproductive defects typical of AMPK compromise in *C. elegans* dauer larvae. Our work provides a further extension in the understanding of how reproductive development must be tightly regulated in response to environmental factors. In addition, we reveal how this critical metabolically responsive enzyme is required for the proper function of small RNA pathways which are necessary for developmental progression and reproductive fitness in *C. elegans*.

## Materials and methods

### Maintenance of *C. elegans* strains

All *C. elegans* strains were grown on nematode growth media (NGM) plates seeded with OP50 *Escherichia coli* bacteria. All strains containing the *daf-2(e1370)* mutation were maintained at the permissive temperature of 15°C to ensure they did not enter dauer.

Bleach synchronization of embryos was performed using sodium hydroxide and sodium hypochlorite solutions, using standard protocols [49]. Bleached embryos were kept in M9 solution for 18 h before being dispensed onto plates as synchronized L1 larvae. For dauer formation, animals were grown at 25°C from the L1 stage.

For strains with the *daf-7* mutation, a mixed population of rollers (containing the *daf-7* rescue transgene) and nonroller animals was maintained, as *daf-7* mutants are often dauer constitutive, especially in an *aak(0)* background and difficult to work with. For dauer induction, this mixed population was bleach synchronized as normal and grown at 25°C. Roller progeny with the *daf-7* rescue did not form dauers, and so after ∼48 h plates were washed and subjected to 1% sodium dodecyl sulfate treatment for 15 min, then re-plated and cultured for another 48 h at 25°C. This resulted in the removal of all nondauer progeny.

All strains used in this study are listed in the following table:

**Table:** 

Strain	Genotype	Source
CB1370	*daf-2(e1370) III*	CGC [50]
MR1000	* daf-2(e1370); aak-1(tm1944) III; aak-2(ok524) X*	[34]
CB4108	*fog-2(q71) V*	CGC [51]
MR3099	* daf-7(1372) III; rrEx979[pMR2197 daf-7::daf-7; rol-6(+)]*	This study
MR3115	* aak-1(tm1944) daf-7(1372) III; csr-1(tor67[gfp::3xflag::csr-1]) IV; aak-2(ok524) X; rrEx979[pMR2197 daf-7::daf-7; rol-6(+)]*	This study
MR3072	*daf-2(e1370); alg-4(ok1041) III; alg-3(tm1155) IV*	This study
MR3066	* daf-2(e1370); aak-1(tm1944); alg-4(ok1041) III; alg-3(tm1155) IV; aak-2(ok524) X*	This study
MR3123	*daf-2(e1370) III; alg-3(tor141[GFP::3xFLAG::alg-3]) IV*	This study
MR3028	* eri-3(tm1361)II; daf-2(e1370) aak-1(tm1944)III; alg-3(tor141[GFP::3xFLAG::alg-3]) IV aak-2(ok524)X*	This study
MR3064	*daf-2(e1370) III; csr-1(tor67[gfp::3xflag::csr-1]) IV*	This study
MR3065	* daf-2(e1370) aak-1(tm1944) III; csr-1(tor67[gfp::3xflag::csr-1]) IV; aak-2(ok524)X*	This study
MR3118	* ieSi64 [ieSi64 [gld-1p::TIR1::mRuby::gld-1 3′ UTR + Cbr-unc-119(+)] II; daf-2(e1370) aak-1(tm1944) III; csr-1(gc029[degron::mCherry::3xflag::HA::csr-1]) IV; aak-2(ok524)X*	This study
MR2409	* daf-2(e1370); aak-1(tm1944) III; eri-1(ok2683)IV; aak-2(ok524)X*	Our lab [52]
MR2374	* eri-3(tm1361)II; daf-2(e1370) aak-1(tm1944)III; aak-2(ok524)X*	Our lab [52]
MR2423	* eri-6(mg379)* I; *daf-2(e1370) aak-1(tm1944)III; aak-2(ok524)X*	Our lab [52]
MR2424	* eri-9(gg106) daf-2(e1370) aak-1(tm1944)III; aak-2(ok524)X*	Our lab [52]

### RNA extraction and Sequencing

For RNA sequencing (RNA-Seq), ∼10 000–20 000 dauer and post-dauer animals, grown on large NGM plates, were harvested per sample. Dauer animals were collected 96 h after being distributed on plates as L1 larvae at 25°C, with all animals containing the *daf-2(e1370)* allele which allows for temperature-induced dauer formation. For post-dauer animals, populations were shifted to 15°C to induce dauer recovery, then collected 96 h afterwards (or 196 h after initial plating). Animals that escaped dauer and began to develop early were removed, primarily 24–48 h after the shift to 15°C. At the time of harvest, any animals that were not in the adult stage were also removed. This ensured a level of synchrony within the post-dauer populations.

For Reverse Transcription quantitative PCR (RT-qPCR) applications, ∼5000 dauer larvae, as well as adult animals for the Taqman assay, grown on medium-sized NGM plates, were harvested per sample. Dauer animals were collected 96 h after being put on plates as synchronized L1 larvae at 25°C, whereas adults were collected ∼96 h after being put on plates as synchronized L1 larvae, with growth at 15°C.

#### Messenger RNA (mRNA) extraction

Total RNA was extracted using Trizol, as described previously [53]. Briefly, 200 µl of Trizol (Ambion TRIzol reagent) was added to pelleted dauer larvae in Eppendorf tubes, followed by four cycles of freeze thawing in liquid nitrogen, and then 1 h of incubation at 4°C. Samples were spun at 13 000 revolutions per minute at 4°C for 10 min before solutions were transferred to a new tube, without disturbing the pellet. Then, 50 µl chloroform was added to each new tube followed by vortexing and 3 min rest at room temperature, and then spinning for 15 min at 4°C. The aqueous layer was transferred to new tubes and the chloroform extraction was repeated. Samples were then treated with 125 µl isopropanol, inverted to mix, and spun at high speeds for 10 min, then washed with 70% EtOH and spun again for 5 min. Solutions were aspirated and then air-dried for 1 h, followed by mixing with 10–20 µl ultrapure water and annealing at room temperature.

RNA concentration was measured using a nanodrop spectrometer, and an aliquot of each sample was run on a 1% agarose gel to confirm presence of 18s and 28s ribosomal RNA bands in order to ensure samples retained integrity. For RNA-Seq samples, RNA was stored at –80°C for up to 3 days before being delivered in a dry ice container to sequencing facility at Institute for Research in Immunology and Cancer (IRIC), located at the Université de Montréal. Sequencing was performed using NextSeq Midoutput with 2 × 75 bp coverage, 150 cycles Paired-End. Three biological replicates were each of the four sample types: *daf-2* dauer, *daf-2* post-dauer, *daf-2; aak(0)* dauer, *daf-2; aak(0)* post-dauer, *daf-2; aak(0); alg-3/4* dauer and *daf-2; aak(0); alg-3/4* post-dauer.

#### Analysis of RNA-Seq data

Primary analysis of RNA-Seq data were carried out by the sequencing facility at IRIC in Montreal, QC, Canada. Briefly, sequences were trimmed for sequencing adapters and low quality 3′ bases using Trimmomatic version 0.35 [54], aligned to reference *C. elegans* genome version WBcel235 with gene annotation from Ensembl version 104, using STAR version 2.7.1a [55]. Gene expression was obtained as a readcount directly from STAR, or computed using RSEM [56] in order to obtain transcript level expression either in TPM or FPKM values for stranded RNA libraries.

For differential gene expression, raw readcounts were filtered for low-read genes, then analysed using DESeq2 v1.48.2 [57]. Shrunken log fold changes were calculated using apeglm or ashr [58, 59]. Pairwise comparisons were carried out between dauer and post-dauer datasets of each genotype, and between *daf-2* and *daf-2; aak(0)* datasets of each developmental stage. Additionally, pairwise comparisons were carried out between *daf-2; aak(0); alg-3/4* and *daf-2; aak(0)* datasets. For differential gene expression analysis, genes were filtered based on *P*-value (<0.05) and fold change (1.5, or log_2_ of 0.58). Normalized readcounts from DESeq2 were used for further data analysis e.g. in heatmaps.

Tissue, phenotype and gene ontology enrichment analysis was carried out using WormBase’s Gene Set Enrichment Analysis [60, 61]. Figures, including Volcano plots, Bubble plots, heatmaps, and Venn diagrams were generated using a combination of R, Microsoft Powerpoint, and online resources [62]. Data analysis, including thresholding and pairwise comparisons were done in R or Microsoft Excel.

#### Small RNA sequencing

1–2 µg of total RNA were treated with polyphosphatase to allow cloning of small RNAs independent of their 5′ phosphate group. Then, for library preparation, the Illumina TruSeq small RNA kit was used with 15 cycles of amplification. Small RNA libraries were size selected on a tris base, boric acid, ethylenediaminetetraacetic acid (TBE) 6% gel. Libraries were sequenced on a HS1500.

Bioinformatic analysis: Adapter sequences, reads shorter than 18 nucleotides (nt), and reads longer than 36 nt were removed using cutadapt. Then, small RNA reads were aligned using STAR against the *C. elegans* genome WS235 [55]. Read counts per genetic element of the Wormbase genome annotation WS235 were calculated using feature counts [63]. Reads were normalised using pseudo-reference with geometric mean row by row [64]. Statistical analysis was performed using Benjamini–Hochberg adjustment [65]

### RNAi experiments

Animals were subjected to RNAi via feeding, as described previously [66]. Bacteria producing double-stranded RNA (dsRNA) against selected genes, from the Ahringer library, were grown in Lysogeny Broth (LB) culture containing ampicillin overnight at 37°C. Then, 300 µl bacterial culture was seeded onto NGM plates containing Isopropyl β-D-1-thiogalactopyranoside (IPTG) and ampicillin. Plates were grown for at least 1 day at room temperature before use in any assays. L1 synchronized animals were seeded onto plates and then grown at 25°C to promote dauer entry, as described. Animals were kept at 25°C for 96 h before being moved to 15°C for subsequent analysis, or collected at 96 h for certain assays.

### Post-dauer fertility assay

Synchronized L1 animals were put onto NGM plates and grown at 25°C to induce dauer. After 96 h of growth, 50 animals per treatment group were singled onto separate plates and grown at 15°C for 1 week to induce post-dauer development, after which fertility was assessed by checking for the presence of F1 progeny. The percentage of fertile worms per replicate was recorded.

### Brood size assay

Post-dauer animals were singled and separated onto individual plates, then allowed to lay eggs. Offspring development was tracked and parents were moved onto new plates as needed. Total number of worms on each plate per original parent was counted. A total of 25 individual animals had their brood size assayed for each experiment.

### Confocal microscopy

Confocal microscopy was performed on a Leica DMI 6000B inverted microscope equipped with a Quorum WaveFX spinning Disc and EM CCD, under various magnifications with and without oil immersion. ZEN Microscopy software was used for both the imaging as well as image processing.

Slide preparation involved using 2% agarose microscopy pads heated through microwaving until molten, then dispensed onto glass slides and covered by a second slide until solidified, at which point the top slide was removed. Animals were washed or picked into 20 mM levamisole, or 500 mM for dauer animals. After paralysis, a cover slip was used to seal the animals. 10× or 63× objectives were used to take images, as needed, with Z-stacking when appropriate. A range of 15 μm with slices at 0.2 μm were used.

Post-processing involved trimming blurry Z-stacks (generally at upper and lower limits of the stack), and creating image subsets to focus on worm bodies (removing empty space). This was followed by deconvolution (constrained iterative) and extended depth of focus to condense stacks. Images were subsequently processed using the application Fiji to determine scale bar length, and trimmed and arranged using Microsoft Powerpoint for compound figures. All images within each figure panel are from the same experimental batch, and any brightness/contrast adjustments on images were carried out uniformly across all presented images.

### Staining of dauer larvae

#### DAPI (4′,6-diamidino-2-phenylindole) staining

Dauer larvae were grown as described. After 96 h, animals were washed into tubes using M9, then the solution aspirated, and 500 µl Carnoy’s solution (60% ethanol, 30% acetic acid, 10% chloroform) was added. Samples were then rotated overnight at 4°C until the following day.

Samples were then washed with PBST (1× PBS with 0.1% Tween 20) three times before being aspirated, and 0.1 mg/ml DAPI was added to each tube. Samples were then rotated at room temperature for 30 min. Subsequently, four PBST washes were performed, with 15 min room temperature incubation with rotation after each wash. Finally, samples were aspirated to leave behind 20 µl liquid, then dispensed directly onto clean glass slides to dry. A cover slip was placed over each sample and sealed with nail polish.

#### Counting germ cells in dauer larvae

Germ cell counts were carried out with DAPI-stained dauer larvae. The germ lines of individual animals were observed under a 63× objective with oil immersion using a confocal microscope. The number of germ cells was counted in each animal, with 25 animals used per genotype or condition for each assay. Samples were imaged immediately following DAPI staining, or stored at 4°C.

#### Counting animals with visible sperm

Samples were imaged using confocal microscopy as described, and stored at 4°C. For determining the percentage of animals exhibiting spermatogenesis, individual animals on each slide were tracked using Zen software, and germ lines of each animal was observed throughout its Z-axis. 50 animals per replicate were examined.

### Mating assay

Males and L4 hermaphrodites/*fog-2* females were placed on cross plates i.e. small NGM plates with a small bacterial lawn in the center. Due to the inconsistent nature of post-dauer development, post-dauer L4 animals were picked based on visible morphology rather than a specific time point as with other assays. For mating, 5 hermaphrodite/females were used with 25 males. After 2 days, hermaphrodites/females were singled onto plates, and observed after 1 week to note the presence of progeny, specifically male progeny. The percentage of fertile animals with progeny was noted. 25 hermaphrodites were used for each sample.

### RT-qPCR

#### For mRNA

Worms were either washed or picked into tubes, then subjected to Trizol RNA extraction. Roughly 1 µg of extracted total RNA per sample was converted to complementary DNA (cDNA) using the High-Capacity RNA-to-cDNA^TM^ Kit per instructions (AppliedBiosystems). RT-qPCR was performed with 1–2 ng of RNA per sample using the 2× SyberGreen qPCR Master-mix (ZmTech Scientifique).

For measurement, the Bio-Rad CFX384 Real-Time 96-well polymerase chain reaction (PCR) qPCR Detection System (Bio-Rad) was used, and the CFX Maestro Software (Bio-Rad) was used to analyse RT-qPCR data. *tba-2* served as a reference gene for calculations. Three biological replicates, sourced from separate collected RNA samples, were performed for each reaction, as well as one negative control replicate using DNAase-free H_2_O instead of cDNA. Fold change calculations were performed using the delta cycle threshold (CT) method.

All primers are listed in Supplementary Table S1.

#### For short interfering RNA 

Specific TaqMan small RNA assays were used to detect short interfering RNA (siRNA) levels. siRNAs were reverse transcribed from total RNA using the TaqMan miRNA Reverse Transcription Kit (Applied Biosystems), as advised by the manufacturer. Then, 2 ng of total purified RNA, and 5× Reverse Transcriptase (RT) primer (Applied Biosystems) for each genetic background/target siRNA were used, with U18 small nucleolar RNA (snoRNA) as the housekeeping control. The levels of target small RNAs were quantified from the cDNA using TaqMan miRNA Assays (Applied Biosystems) using 20× probes specific for each siRNA. A Bio-Rad CFX384 Real-Time 96-well PCR qPCR Detection System (Bio-Rad) coupled with the CFX Maestro Software (Bio-Rad) was used to analyse RT-qPCR data. Three biological replicates were performed for each RT-qPCR reaction, with one additional replicate using DNAase-free H_2_O instead of cDNA. Fold change calculations were performed using the delta CT method.

Taqman primers are listed in Supplementary Table S1.

### Auxin-inducible degron (AID) experiments

Auxin treatment was done on NGM plates supplemented with indole 3-acetic acid (IAA, or auxin, Sigma–Aldrich). Then, 1 M stock solution of IAA was created, and appropriate volumes added to molten NGM solution for plates with a working concentration of 100 µM or 1 mM.

For auxin-induced degradation of CSR-1, animals were only put onto auxin supplemented plates during the dauer stage (outlined in Supplementary Fig. S5B). Animals were first bleach-synchronized and grown on regular NGM plates. Worms were then washed onto auxin plates after ∼46 h at 25°C, before they entered dauer. After 48 h of growth at 25°C, animals were either harvested for DAPI staining or RNA extraction, or washed and put onto standard NGM plates for growth at 15°C for post-dauer fertility analysis. Some CSR-1::AID animals were grown on standard NGM plates without auxin throughout the 96 h as a negative control.

### *In vitro* transcription of 26G RNAs

Protocol for transcription of 26G RNAs was adapted from similar techniques described previously [67]. The experimental workflow is outlined in Supplementary Fig. S7D. All primers are listed in Supplementary Table S1.

#### double-stranded DNA (dsDNA) template preparation

First, for each 26G RNA that is to be generated, four DNA oligonucleotides were obtained, ordered through IDT. Primers were dissolved in ultrapure water to a 1 mM concentration. The two pairs of oligonucleotides must be complementary, and the first pair must have the sequence for a T7 RNA Polymerase promoter (e.g. GGTAATACGACTCACTATA, 19nt) followed by the 5′ to 3′ sequence of the 26G RNA of interest. The second oligonucleotide pair must similarly have the T7 promoter in the same direction, but the 26G RNA sequence must be reversed i.e. it must have the 3′ to 5′ strand. For each oligonucleotide pair, 2 µl of each was put into solution with 46 µl TE buffer, for a total of 50 µl, and then annealed at 95–100°C for 3 min, then allowed to cool at room temperature for several minutes. The dsDNA template was then used immediately, or alternatively stored at −20°C.

#### *In vitro* transcription

Transcription carried was out using standard procedures. Each separate reaction generated one strand of 26G RNA. Two separate reactions need to be run to generate both strands.

For a 50 µl final solution, the following mixture was set up:

- 5 µl of 10× NTPs (Nucleoside Triphosphate)
- 5 µl of 10× T7 RNA polymerase buffer
- 2.5 µl of 5× DTT (Dithiothreitol)
- 22.5 µl water

1 µl of RNase Inhibitor (New England Biolabs) was added and placed in a 37°C heat block for 5 min. Subsequently, 10 µl of the annealed dsDNA solution was added followed by 2 µl of T7 RNA Polymerase. After gentle mixing, the reaction was placed in a 37°C heat block for 1 h. Following this, an additional 2 µl of T7 RNA Polymerase (New England Biolabs) was added and the reaction returned to the heat block for 1 h. Finally,1 µl DNase (New England Biolabs) was added and returned to 37°C for 15 min.

At this point, RNA can be extracted and purified through any desired method. To each solution, one volume of buffer-saturated phenol was added followed by vortexing and spinning. The top layer was separated, and one volume chloroform was added, followed by another vortex and spin. The top layer was separated and 2 volumes of ice-cold EtOH, as well as 1/10 volume 5 mM sodium acetate, was added. Samples were precipitated in −20°C for 1 h, then spun down at 4°C at high speed for 20 min. Samples were aspirated, with care taken not to disturb the bottom of the tube. 70% EtOH was added and samples were washed, spinning at max speed at 4°C for 5–10 min. Samples were aspirated, removing as much liquid as possible, and allowed to air dry for 1 h, then dissolved in ultrapure water and their concentration checked with a nanodrop.

For generating dsRNA, equal concentrations of sense and antisense RNA were combined, then added to one half volume of TE buffer, and annealed at 95–100°C in a heat block for 5 min. Samples were cooled room temperature for 30 min before using. Samples can be run on a high concentration (2%–2.5%) agarose gel, and bands should appear lower than the dsDNA template (Supplementary Fig. S7E).

### RNA soaking

#### Total RNA soaking

RNA was extracted from desired *C. elegans* strains and put into solution with M9 and concentrated HB101 bacteria, with RNA concentrations in solution ranging from 100–200 ng/µl, as available. For soaking, bleach-synchronized animals were allowed to hatch into M9 overnight, and then pelleted to remove liquid. Then, 20 µl of RNA solution was added to each worm sample in Eppendorf tubes, and then tubes were placed on a rotator at 25°C for 96 h. Animals were then harvested and used for further experiments. For imaging, a small aliquot was placed onto NGM plates, then animals were picked into agarose slides and imaged, as described above. For western blot, samples were washed thoroughly before being prepared for use in western, described below. Experimental flow for soaking outlined in Supplementary Fig. S7A. See Wong *et al*. (2024) for further details [68]

#### RNA soaking

Procedure as above, except one or more 26G RNA solutions were used in place of total RNA. Concentrations of ∼200 ng/µl were used for all samples. For the 26G RNA cocktail solution, equal concentrations of all three RNAs were mixed.

### Western blot

Collected worm samples in PBST were pelleted and left in a solution of ∼20 µl and frozen at −20°C. Samples were thawed, then flash-frozen in liquid nitrogen followed by boiling in a 100°C heat block. This was performed 3–4 times as needed, until samples were no longer viscous. Samples were briefly vortexed and centrifuged, then loaded onto 6% sodium dodecyl sulphate–polyacrylamide gel electrophoresis gels along with a ladder.

Western blots were initially run at 85 V for the stacking gel, then at 100–120 V for the resolving gel, as needed. After membrane transfer, Ponceau’s solution was used to assess levels of protein on the membrane. Membranes were then cut based on protein size in order to obtain one half with the green fluorescent protein (GFP) signal (>75 kDa) and one half with the α-tubulin signal (<75 kDa). After 1 h of blocking with 5% milk powder solution dissolved in PBST, membranes were incubated with anti-GFP (produced in lab, 1:1000 ratio) or anti-α-tubulin (MilliporeSigma, 1:2000 ratio) antibodies overnight. The following day, membranes were washed three times with PBST, 10–15 min each wash, and then incubated with secondary antibodies (anti-Rabbit, 1:1000 for GFP and anti-Mouse, 1:2000 for α-tubulin) for 2 h. Afterwards, three PBST washes, 15 min each time, were carried out.

For imaging, membranes were soaked with 1 ml of Clarity Western ECL Substrate for ∼1 min, sandwiched between clear plastic sheets. Imaging was carried out using the MicroChemi (DNR Bio Imaging Systems) and GelCapture software (version 7.0.18), with exposure times ranging from 0.5 to 20 s as needed. For capturing ladder images, an initial image of the blot was taken with the desired exposure time. Bright light was then turned on, and the region of the gel containing the ladder was outlined using the software, followed by image contrast adjustment on that section to make the ladder visible. The resulting composite was then saved.

### Statistical analysis

For post-dauer fertility, RT-qPCR fold change, % animal assays and western quantification blot etc., all statistical analysis was performed using GraphPad Prism. All assays used three biological replicates. The specific statistical test, along with *P*-value significance and n-values, are listed in the legends of each figure.

## Results

### RNA sequencing of AMPK-deficient dauer and post-dauer larvae reveals widespread changes in gene expression

*Caenorhabditis elegans* larvae lacking AMPK exhibit numerous reproductive and developmental defects both during and following transit through the dauer stage, including germline hyperplasia, premature spermatogenesis, and post-dauer sterility (Supplementary Fig. S1A and B) [21, 34]. The notable differences in metabolism, survival, development, and reproductive capacity in AMPK mutants are difficult to explain, but could potentially be accounted for through a dramatic misregulation of gene expression. Nevertheless, it is unclear how these regulatory pathways are impacted by AMPK function, and how a single metabolic regulator could contribute to such a diverse spectrum of processes that go awry in its absence.

To determine if, or how, the loss of AMPK influences gene expression to cause these phenotypes, we performed gene expression analysis with dauer and post-dauer animals, comparing a control strain, *daf-2(e1370)* that possessed wild-type AMPK function, with an AMPK null mutant (with loss-of-function alleles for *aak-1* and *aak-2*) which also contained the same *daf-2* mutation. This *daf-2* allele is dauer-constitutive at temperatures of 25°C, but behaves essentially as wild type at permissive temperatures (15°C), allowing us to toggle between dauer and post-dauer development by switching temperature [69, 70].

**Figure 1.**
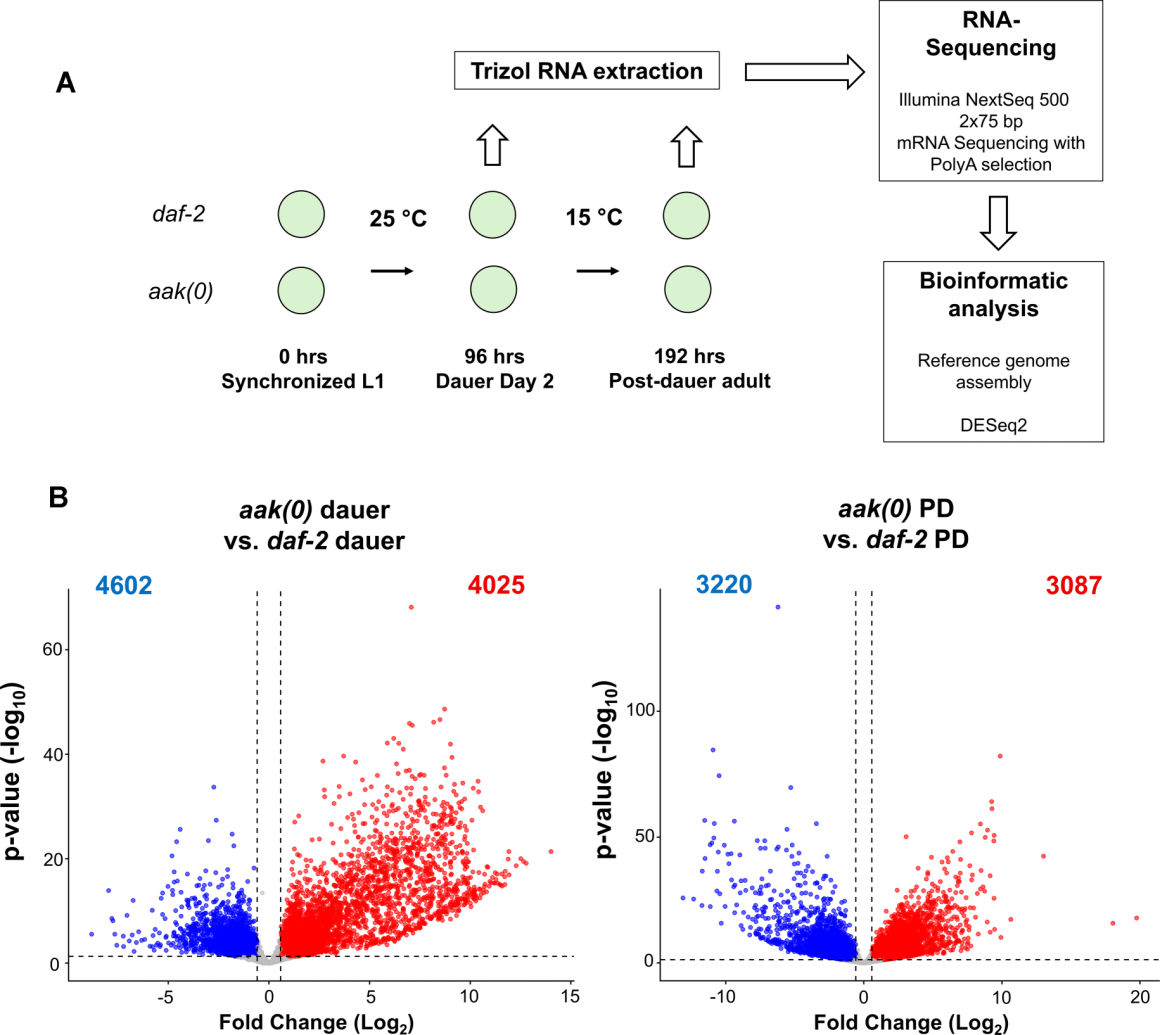
RNA Sequencing of *aak(0)* and *daf-2* dauer larvae and post-dauer (PD) adults reveals widespread changes in gene expression. (**A**) Schematic diagram describing the protocol for RNA extraction and sequencing from indicated samples. *daf-2* and *aak(0)* dauer larvae were harvested for RNA-Seq analysis 96 h after growth at 25°C. Samples of *daf-2* and *aak(0)* post-dauer animals were harvested 96 h after the transition to 15°C. Approximately 10–20, 000 animals, cultured on large plates, were harvested per replicate, three biological replicates for each condition. Total RNA from all samples was extracted using standard Trizol extraction, then sent for messenger RNA (mRNA) sequencing and subsequent bioinformatic analysis. Sequences were aligned to the reference genome and normalized using DESeq2. Further bioinformatic analysis was done in-house using Microsoft Excel and R. (**B**) Differential gene expression in *aak(0)* dauer larvae (left) and post-dauer (right) animals compared to *daf-2* controls is expressed in Volcano plots generated by R. Red and blue dots represent increased and decreased expression, respectively, based on log_2_ fold change of *aak(0)* values compared to *daf-2* control. Expression is graphed as a function of *P*-value, and grey dots indicate genes that were below the cutoff threshold for significance (*P*-value >0.05) and fold change (FC) (log_2_ FC between 0.58 and −0.58). All animals were assessed in a *daf-2* genetic background.

Pairwise comparisons were performed between dauer and post-dauer animals of the same genotype, as well as between *daf-2* control and *daf-2; aak-1; aak-2* mutants [hereafter referred to simply as *aak(0)*] of the same stage (Fig. 1A). Data were further thresholded based on *P*-values and minimum mean expression to eliminate noise, as well as fold change for pairwise comparisons. Initial analysis of the RNA-Seq data revealed a large number of differentially expressed genes (DEGs) between control and AMPK mutant animals in both the dauer and post-dauer stage (Fig. 1B), suggesting widespread misregulation of gene expression due to the absence of AMPK in these animals.

We validated our RNA-Seq results through quantitative RT-qPCR of two highly enriched genes that were demonstrated to show differential expression between *daf-2* and *aak(0)* mutants based on our sequencing data (Supplementary Fig. S1C). Furthermore, to confirm that this altered gene expression in AMPK-deficient animals does not result from the *daf-2* mutation but is dauer-specific, we performed similar validation assays in animals that lack *daf-7*, which results in temperature-sensitive dauer formation in a manner similar to *daf-2*, but by impinging on a separate genetic pathway [71]. Comparing RT-qPCR expression data between the *daf-7* and *daf-7; aak(0)* strains and their *daf-2* counterparts, we saw similar changes in gene expression (Supplementary Fig. S1D), suggesting the transcriptomic changes observed in *aak(0)* animals are not unique to animals that enter dauer due to the *daf-2* mutation.

Consistent with our previous findings, the physiological analysis of *aak(0)* dauer larvae indicates these mutants undergo premature spermatogenesis as well as germline hyperplasia (Supplementary Fig. S1A and B), both of which are consistent with AMPK mutant dauers abnormally initiating reproductive development despite the dauer signals that instruct them to maintain a quiescent state. We thus chose to focus on the changes to reproductive gene expression to better understand how the various reproduction-associated phenotypes arise in AMPK mutants and how the compromise of small RNA homeostasis impacts the expression of these genes.

### Reproductive gene expression is prematurely induced in AMPK mutant dauer larvae

**Figure 2.**
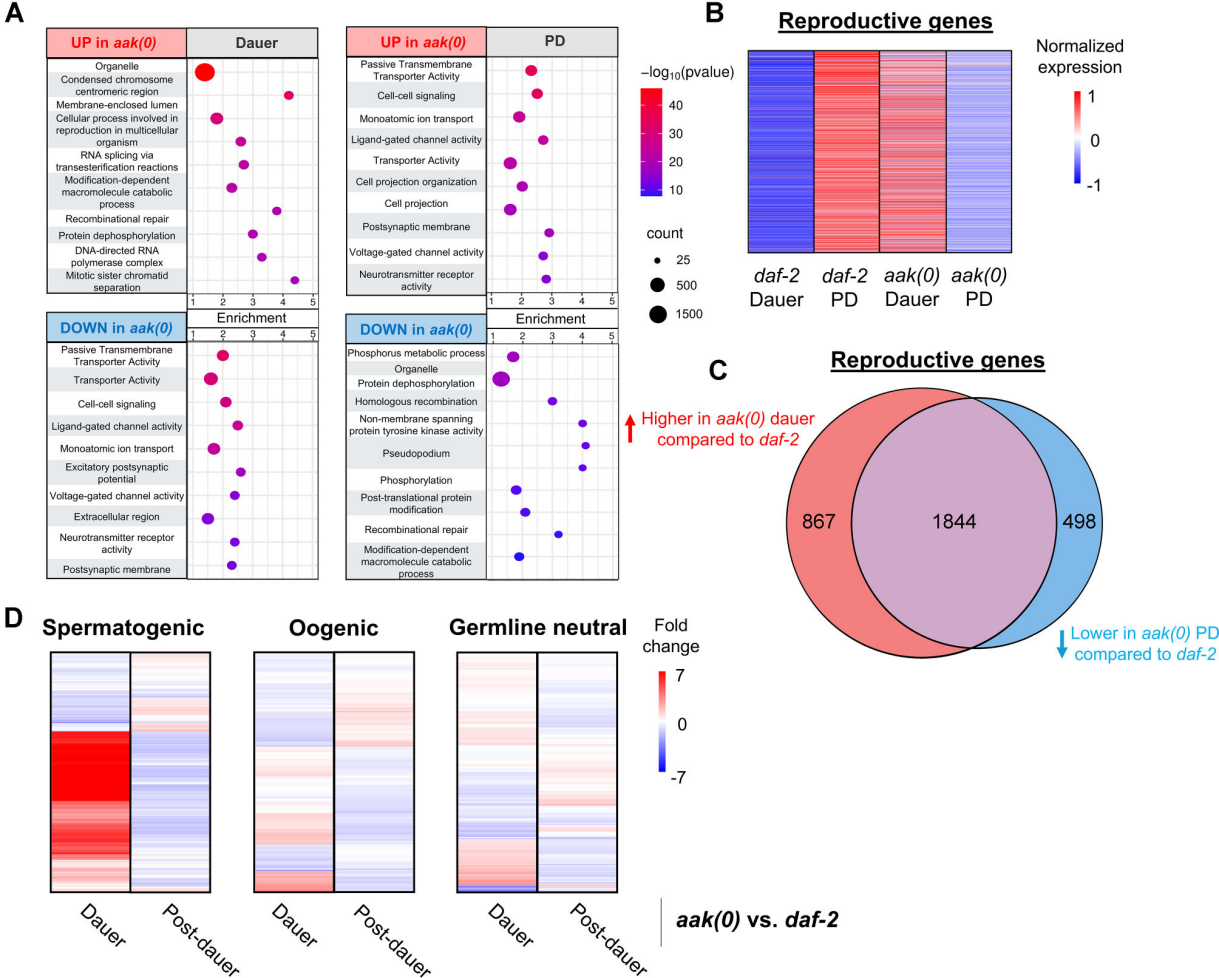
AMPK mutant animals exhibit widespread misregulation of reproductive genes. (**A**) Bubble plots depicting the most enriched Gene Ontology (GO) terms in *aak(0)* versus *daf-2* control DEG set, ranked by significance based on *P*-value. Left and right graphs depict dauer and post-dauer (PD) comparisons, respectively. Top graphs depict genes increased in *aak(0)* mutants compared to controls, while bottom graphs depict genes that decreased. Size of bubbles indicates the number of genes in their respective categories that were enriched in the indicated dataset. GO enrichment was conducted using the Wormbase Gene Set Enrichment Analysis using a q-value threshold of 0.1 [60, 61]. (**B**) Heatmap of the expression of 3708 reproductive genes, highlighting differential expression between *aak(0)* dauer and post-dauer animals compared to *daf-2* controls. Reproductive genes were selected from all DEGs in the *aak(0)* versus *daf-2* comparisons for both dauer and post-dauer, and identified as “reproductive” genes based on associated GO terms. Data represent normalized transcripts per million values for each gene, with higher relative expression in red and lower in blue. See Supplemental Raw Data for list of genes used in analysis. (**C**) Venn diagram of reproductive genes in two categories. Left (red): Increased expression in *aak(0)* dauer larvae compared to *daf-2* controls. Right (blue): Decreased expression in *aak(0)* post-dauer animals compared to *daf-2* controls. A total of 1949 genes were present in both subsets. (**D**) Heatmap of spermatogenic, oogenic, and germline-neutral gene expression in *daf-2* and *aak(0)* dauer and post-dauer animals. Data compare log_2_ fold change between *aak(0)* versus *daf-2* samples. Gene lists obtained from Ortiz *et al*. (2014) [72]. All animals were assessed in a *daf-2* genetic background.

Although the loss of AMPK disrupts cellular homeostasis at multiple levels during periods of energy stress, one of the predominant phenotypes observed during dauer development is a pronounced germline hyperplasia that occurs just prior to dauer entry, followed by a penetrant sterility observed in post-dauer recovered AMPK mutant adults [21, 28]. To determine if this misregulation might be dependent on the observed abnormalities in gene expression, we performed enrichment analysis of our RNA-Seq data by comparing the dauer stage *daf-2* and *aak(0)* RNA datasets [60, 61]. From these comparisons, we noted that genes associated with reproduction, meiosis, and germline function were all aberrantly regulated in the *aak(0)* dauer larvae (Fig. 2A, top left). Tissue-specific enrichment analysis also revealed an increase in genes associated with the categories ‘male’, ‘germ line’, and ‘reproductive organ’ (Supplementary Fig. S2A). This finding indicates that reproductive pathways are aberrantly active in *aak(0)* dauer larvae compared to the control *daf-2* animals, which are normally quiescent in the dauer stage. The increase in germ cell numbers in *aak(0)* dauer larvae likely contributes to some of these changes in gene expression, yet the extent of these shifts, particularly in terms of male or spermatogenic genes, nevertheless suggests that the loss of AMPK signaling leads to a misregulation of reproductive programs.

We noted an inverse gene expression pattern when comparing the post-dauer datasets to the dauer RNA-Seq data. There was an overlap between GO terms enriched in genes with increased expression in dauer (Fig. 2A, top left) and decreased in post-dauer (bottom right), and vice versa. Many of the genes increased in *aak(0)* dauer appeared to be associated with reproductive functions, whereas these were then decreased in the post-dauer. Indeed, when considering the mean expression of differentially expressed reproductive genes, rather than the fold change comparisons, we note similar high levels of expression are shared between the *daf-2* post-dauer and *aak(0)* dauer (Fig. 2B). Conversely, *daf-2* dauer and *aak(0)* post-dauer have similar low levels of expression for these same genes. There is an overlap of ∼1844 reproductive genes between the subset elevated in *aak(0)* dauer and decreased in *aak(0)* post-dauer (Fig. 2C). This suggests that the *aak(0)* mutants execute these reproductive programs earlier than their *daf-2* control counterparts, specifically during the dauer stage.

To confirm whether there truly is a dysfunction of reproductive programs in *aak(0)* dauer larvae, we quantified the differential expression of specific spermatogenic, oogenic, and sex-neutral germline genes in the dauer and post-dauer stages. We obtained a list of genes in these categories [72] and compared fold changes between *aak(0)* and control *daf-2* dauer and post-dauer animals (Fig. 2D). The data revealed a sharp contrast between the different gene sets: a large percentage of spermatogenic genes had greatly increased expression in *aak(0)* dauer larvae, but most were decreased in the post-dauer animals, suggesting spermatogenic gene expression is very strongly affected by the absence of AMPK. By contrast, the changes to oogenic and germline-neutral gene expression were less dramatic, but many of these genes nevertheless showed increased expression in *aak(0)* dauer larvae. Notably, we saw strong inverse expression of these genes in the post-dauer, mimicking the overall expression patterns we see in the reproductive gene list (Fig. 2B). These comparisons lend further credence to the hypothesis that there is a disruption of reproductive gene expression in *aak(0)* dauer larvae, such that many of these genes are prematurely expressed during dauer, and then reduced in post-dauer.

We next wondered whether the increased reproductive gene expression noted in *aak(0)* dauer animals correlated with the gene expression changes observed in control post-dauer animals undergoing reproductive development. We performed a three-way analysis, comparing the genes with increased expression in *aak(0)* dauer with the subset that are increased in the *daf-2* post-dauer compared to *daf-2* dauer, as well as with the subset of genes decreased in *aak(0)* post-dauer animals (Supplementary Fig. S2B). This comparison revealed a significant overlap, comprising 1970 genes, across the three subsets (Supplementary Fig. S2B, grey). This subset is comparatively enriched in *aak(0)* dauer larvae and in *daf-2* post-dauer animals, while they are simultaneously reduced in this stage in the *aak(0)* adults. Gene ontology analyses revealed that this large subset of overlapping genes is associated with reproductive phenotypes (Supplementary Fig. S2C).

Given the significant overlap of genes, we conclude that the absence of AMPK results in dauer animals adopting a transcriptomic profile similar to that of typical post-dauer reproductive adults, essentially causing the mutants to skip the dauer stage entirely, at least from a reproductive perspective. This premature expression of reproductive genes likely contributes to the observed germline hyperplasia and premature spermatogenesis, as well as the subsequent post-dauer sterility. The gene activation occurs prematurely, but it does not persist, as the expression of these genes drops to below a baseline level in the *aak(0)* post-dauer stage. This punctuated expression therefore causes a miscoordination of germline and somatic development resulting in a failure to establish reproductive competence.

Our comparative analyses also revealed further subsets of genes that are misregulated in the mutant background. We identified 66 genes that are differentially expressed in both *aak(0)* dauer and post-dauer compared to *daf-2* (Supplementary Fig. S2B, dark yellow), but not altered in the *daf-2* dauer versus post-dauer analysis, suggesting these genes are specifically affected by the absence of AMPK, but are not developmental stage-specific. Furthermore, there are 1359 genes that are differentially expressed in *aak(0)* dauer versus *daf-2* dauer, and also in *daf-2* post-dauer versus dauer, but not in *aak(0)* post-dauer versus *aak(0)* dauer (Supplementary Fig. S2B, pink). These appear to be genes associated with reproduction, but unlike the larger subset of 1970 genes, they do not decrease in the *aak(0)* post-dauer. The impact of these genes remains unclear, but their unmodulated expression in *aak(0)* post-dauer could contribute significantly to the observed AMPK mutant phenotypes. Finally, we noted 630 genes that are enriched specifically in *aak(0)* dauer larvae compared to controls, but not in *daf-2* post-dauer compared to dauer (Supplementary Fig. S2B, red). These appear to be reproductive genes based on tissue enrichment, but GO enrichment indicates there are a number of genes associated with ribosomes and protein synthesis (Supplementary Fig. S2D), rather than reproductive functions as seen in larger subset of shared genes. This small subset of genes that are specifically enriched in *aak(0)* dauer may collectively contribute to any one, if not several of the AMPK mutant phenotypes, particularly those associated with aberrant cell growth and proliferation in the germ line.

The dramatic germline hyperplasia we observe in *aak(0)* dauer larvae could potentially account for some of the observed changes in reproductive gene expression. To verify this possibility, we normalized our data to a prominent germ cell marker gene, *pgl-1* by subtracting the *pgl-1* fold change from the fold changes of our genes of interest. We compared the adjusted fold change of reproductive genes and saw that, while the values were indeed lower than the original comparison, there nevertheless remained a large proportion of reproductive genes in the *aak(0)* dauer larvae that had increased expression compared to the wild type (Supplementary Fig. S2E). Therefore, the increased number of germ cells in these mutants cannot fully explain the increase/change in reproductive gene expression.

### The loss of AMPK has a dramatic impact on the expression of small RNA pathway components during the dauer stage

Previously, we showed that compromise of a select group of small RNA pathway components partially suppresses the post-dauer sterility associated with AMPK mutants [34]. Given the widespread changes in gene expression observed in AMPK-deficient mutants, particularly with respect to germ line and reproductive genes, it is highly plausible that the misregulation of one or more RNAi pathways that occurs in the absence of AMPK may be responsible for these transcriptomic changes and their consequent reproductive defects.

**Figure 3.**
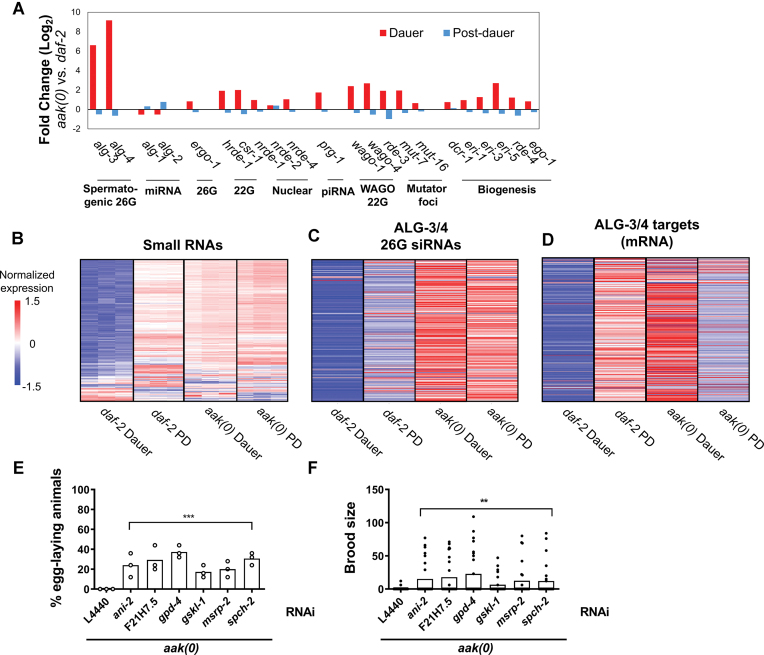
Gene expression of RNAi pathway components is increased in AMPK dauer larvae. (**A**) Bar graphs depicting fold changes of RNAi pathway components in *aak(0)* dauer and post-dauer (PD) animals compared to *daf-2* controls. Red bars indicate log_2_ fold change in dauer, blue bars represent post-dauer. Genes categorized based on function or family and are listed in Supplementary Table S2 [73]. (**B**) Heatmap depicting normalized expression of all small RNAs in *daf-2* and *aak(0)* dauer and post-dauer (PD) animals, based on small RNA-Seq data. Data represent normalized transcripts per million values for each gene, with higher relative expression in red and lower in blue. (**C**) Heatmap specifically depicting normalized expression of ALG-3/4 26G small RNAs in *aak(0)* and *daf-2* dauer and post-dauer animals. (**D**) Heatmap of the mRNA expression of gene targets of ALG-3/4 spermatogenic 26G RNAs, selected based on small RNAs that were enriched in *aak(0)* dauer larvae. Data represent normalized transcripts per million values for each gene, with higher relative expression in red and lower in blue. See Supplemental Raw Data for list of genes used in analysis. (**E, F**) Post-dauer fertility and brood size of *aak(0)* mutant animals following RNAi against select enriched ALG-3/4 small RNA targets. L4440 empty vector serves as a control. Animals were grown at 25 °C from synchronized L1s for 96 hours before being singled onto plates. After approximately a week, fertility of each animal was assessed and the total % of egg-laying animals per sample was recorded. For brood size assays, animals were singled and the brood size of each individual animal was measured. Post-dauer fertility data represent three independent trials, with the mean represented by columns and values for individual trials indicated by small circles. n=50 for each trial of post-dauer fertility, n=25 for brood size assays. ***p < 0.001, **p < 0.01 using one-way ANOVA, all values compared to those from the L4440 control. All animals were assessed in a *daf-2* genetic background.

The importance of small RNAs during reproductive development has been well-characterized in *C. elegans* [41, 42, 46]. We questioned whether changes in RNAi pathway gene expression could influence levels of small RNAs, and whether that in turn affected the expression of reproductive genes. We selected a number of pivotal small RNA pathway components (Supplementary Table S2) [73] and compared their expression levels in our RNA-Seq dataset. Almost all of their transcripts were dramatically increased in the *aak(0)* dauer dataset compared to *daf-2* controls (Supplementary Fig. S3A). Most Argonaute family members, which are responsible for small RNA function [73], show a much higher expression level in *aak(0)* dauer, with the spermatogenic 26G RNA Argonautes *alg-3* and *alg-4* showing the highest fold change increase (Fig. 3A). Factors involved in siRNA biogenesis such as the *eri* genes also showed increased levels, suggesting levels of small RNAs themselves were misregulated.

Conversely, small RNA pathway genes had reduced expression in the post-dauer stage compared to controls, similar to our observations of reproductive genes (Fig. 2B). This could suggest that elevated levels of siRNA activity, particularly 26G spermatogenic RNAs, are associated with the abnormal onset of a wave of gene expression associated with reproductive development that occurs in these animals. Indeed, many classes of siRNAs are required to direct various germline functions [44]. If this development occurs prematurely during the dauer stage in AMPK mutants, then expression of these genes may no longer be required or programmed in the post-dauer adult.

We carried out small RNA-Seq to measure the levels of small RNAs in *daf-2* and *aak(0)* dauer and post-dauer larvae. The analysis measured levels of miRNAs, 22G and 26G short interfering RNAs (siRNAs), piwiRNAs (piRNAs), and others. Expression of small RNAs is low in control (*daf-2)* dauer animals, but increased in the post-dauer, when they are reproductively active, which was expected (Fig. 3B). To our surprise, we saw that the small RNA profile of *aak(0)* dauer larvae was greatly increased and looked much more like that of wild-type post-dauer animals. This is consistent with our transcriptomic data, which suggests that in *aak(0)* dauers, reproductive genes at least adopt an expression profile similar to that of adult animals. However, contrary to our observations of the transcriptome, the levels of small RNAs remain abnormally high in *aak(0)* post-dauer, potentially due to misregulated small RNA clearance following their expression, although this remains to be confirmed.

There are a variety of small RNA pathways that target genes in different tissues throughout reproductive development (Supplementary Fig. S3B), and we wondered whether specific classes of small RNAs were driving the differences observed in our sequencing data. The spermatogenic Argonaute genes, *alg-3* and *alg-4*, had the highest fold change increase in *aak(0)* dauer larvae (Fig. 3A). Looking at the small RNA-Seq data, we similarly saw an increase in the levels of most ALG-3/4 associated 26G siRNAs as well (Fig. 3C). This was also true for other classes of siRNAs, including CSR-1 22G RNAs (Supplementary Fig. S3C). There likely exists some mechanism upstream of small RNA biogenesis that is disrupted by the absence of AMPK, thereby leading to such a dramatic increase in small RNA expression in *aak(0)* dauer animals. Notably, the miRNA Argonautes *alg-1* and *alg-2* are among the few small RNA-associated genes with reduced expression in *aak(0)* dauer (Fig. 3A), and miRNAs were also the only class of small RNAs that had reduced expression based on our small RNA-Seq.

The increase in the steady state levels of *alg-3* and *alg-4* transcripts is particularly notable. These Argonautes act in a functionally redundant manner during the processing of spermatogenesis-specific 26G siRNAs [46]. Enrichment of genes in *aak(0)* dauer larvae indicates there is an increase in the expression of genes associated with ‘male’ tissues (Supplementary Fig. S2A), and we saw that spermatogenic gene expression appeared uniquely impacted by the absence of AMPK (Fig. 2D). Indeed, when looking at how these siRNAs may be influencing gene expression, we noted that many 26G siRNA targeted transcripts were more abundant in *aak(0)* dauer larvae (Fig. 3D). This reflects previous observations that ALG-3/4-associated 26G siRNAs can promote transcription of their targets [74].

We analysed the fold change differences of the ALG-3/4 26G RNAs between *daf-2* controls and *aak(0)* mutants, based on our small RNA-Seq experiments, and compared this with the fold change of their cognate genes based on our mRNA-Seq. We saw a positive correlation, whereby most 26G RNAs have increased expression, as do their target genes (Supplementary Fig. S3D). This likely contributes to the premature spermatogenesis that is observed in *aak(0)* dauer larvae [21], highlighting a link between elevated small RNA levels and the observed developmental defects typical of these mutants.

Curiously, while the expression of siRNA target genes decreases, levels of the siRNAs themselves do not decrease in post-dauer *aak(0)* animals (Fig. 3B and C). It is unclear whether this occurs at the level of synthesis or stability, so it is possible that the pathways involved in small RNA degradation are affected in these post-dauer mutants. However, the gene expression patterns across our datasets for “RNAi genes” (Supplementary Fig. S3A) correlate positively with the expression of “reproductive genes” (Fig. 2B), with both gene categories showing increased expression in *aak(0)* dauer and *daf-2* post-dauer. While we suspect the premature reproductive development that occurs in *aak(0)* dauer larvae stems at least partially from the effect of elevated subset of small RNA populations, it is less clear what genes these small RNAs are targeting in the post-dauer stage of these animals.

Given the dramatic increase in ALG-3/4 associated 26G RNAs, we wondered whether the aberrant regulation of their targets could contribute to the post-dauer sterility observed in AMPK mutants. We compared our list of 1970 reproductive genes that are dynamically regulated in AMPK mutants (Supplementary Fig. S2B) with the list of ALG-3/4 26G RNA targets, and found more than half of the 26G target genes are affected by the absence of AMPK (Supplementary Fig. S3E). GO enrichment of this subset of 215 genes revealed they are associated with terms such as phosphorylation and phosphorus metabolic process, showing some overlap with the subset of genes upregulated in *aak(0)* dauer animals (Fig. 2B, top left). Specific genes in this subset include members of the *gskl* family, which are sperm-specific protein kinases that have a role in sperm motility [75]. Their premature expression and subsequent downregulation in AMPK mutants most likely impacts fertility in these mutants.

To determine if any of the upregulated genes were functionally contributing to AMPK mutant phenotypes, we performed an RNAi survey against the most enriched 26G targets that were determined from our RNA-Seq analysis and identified several genes that, when subjected to RNAi, could partially suppress the post-dauer sterility of the AMPK mutants and partially restored brood size (Fig. 3E and F). Two of these genes include *ani-2*, an anillin homolog that contributes to embryonic viability [76], and *spch-2*, associated with sperm chromatin [77]. Nevertheless, no single gene-specific RNAi was sufficient to fully restore post-dauer fertility in the AMPK mutants. This may be the result of variable or incomplete RNAi efficiency, which can be both gene or context specific [78]. Alternatively, it is possible that loss of a single target is insufficient to suppress the numerous phenotypic changes that occur as a result of the loss of AMPK. Given that nearly 2000 reproductive genes are misregulated in *aak(0)* dauer larvae, it is more likely that a combination of a vast spectrum of misregulated genes and pathways contribute collectively to the reproductive defects that culminate in sterility in the AMPK mutants.

The simultaneous increase in the levels of both 26G small RNAs and their target mRNAs could indicate a positive upregulation of the targets via ALG-3/4 activity [74], but it may also reflect a feedback loop whereby an increase in the mRNA levels of the target genes leads to increased production of associated siRNAs derived from those loci. To determine if one, or both, of these scenarios was occurring in these mutants, we verified the levels of several 26G siRNAs using Taqman RT-qPCR to validate our small RNA sequencing data, but also to confirm if there was a bias towards mRNA-derived 26G RNAs. From these data, we noted that in *aak(0)* mutants compared to *daf-2* controls there is a significant increase in 26G RNAs derived from exon and intron coding regions (T27A3.3, F35E2.5, K10B2.5) but also from intergenic regions (C06E4.5) and pseudogenes (Y75D11A.1; Supplementary Fig. S3F; see Supplementary Table S1 for primer sequences). These data are consistent with our sequencing results, and furthermore suggest the widespread increase in small RNAs that we see in AMPK mutants is not solely due to an increase in mRNA levels, as even siRNAs derived from non-mRNA loci are elevated. Notably, when we examine the expression levels in *daf-2; aak(0); alg-3/4* mutants, we see a decrease in the levels of certain 26G RNAs that are known to be ALG-3/4 targets (T27A3.3, F35E2.5, K10B2.5), but not in other 26G RNAs that are not associated with ALG-3/4. This is consistent with ALG-3/4 Argonaute activity contributing to the stability and/or biogenesis of its associated 26G RNAs, but not other classes of 26G RNAs.

### AMPK is required to block untimely ALG-3/4 activity and spermatogenesis during the dauer stage

The inappropriate regulation of small RNAs and their interacting proteins are likely responsible for many of the reproductive defects that arise in AMPK mutants. We not only saw a notable increase in the mRNA levels of the spermatogenic Argonautes *alg-3* and *alg-4*, but we also observed a strong correlation between spermatogenic 26G RNAs and their target gene expression in *aak(0)* dauer larvae. We thus wanted to determine if the misregulation of the ALG-3/4 26G spermatogenic RNAs and their cognate Argonautes played any specific role in the reproductive phenotype.

**Figure 4.**
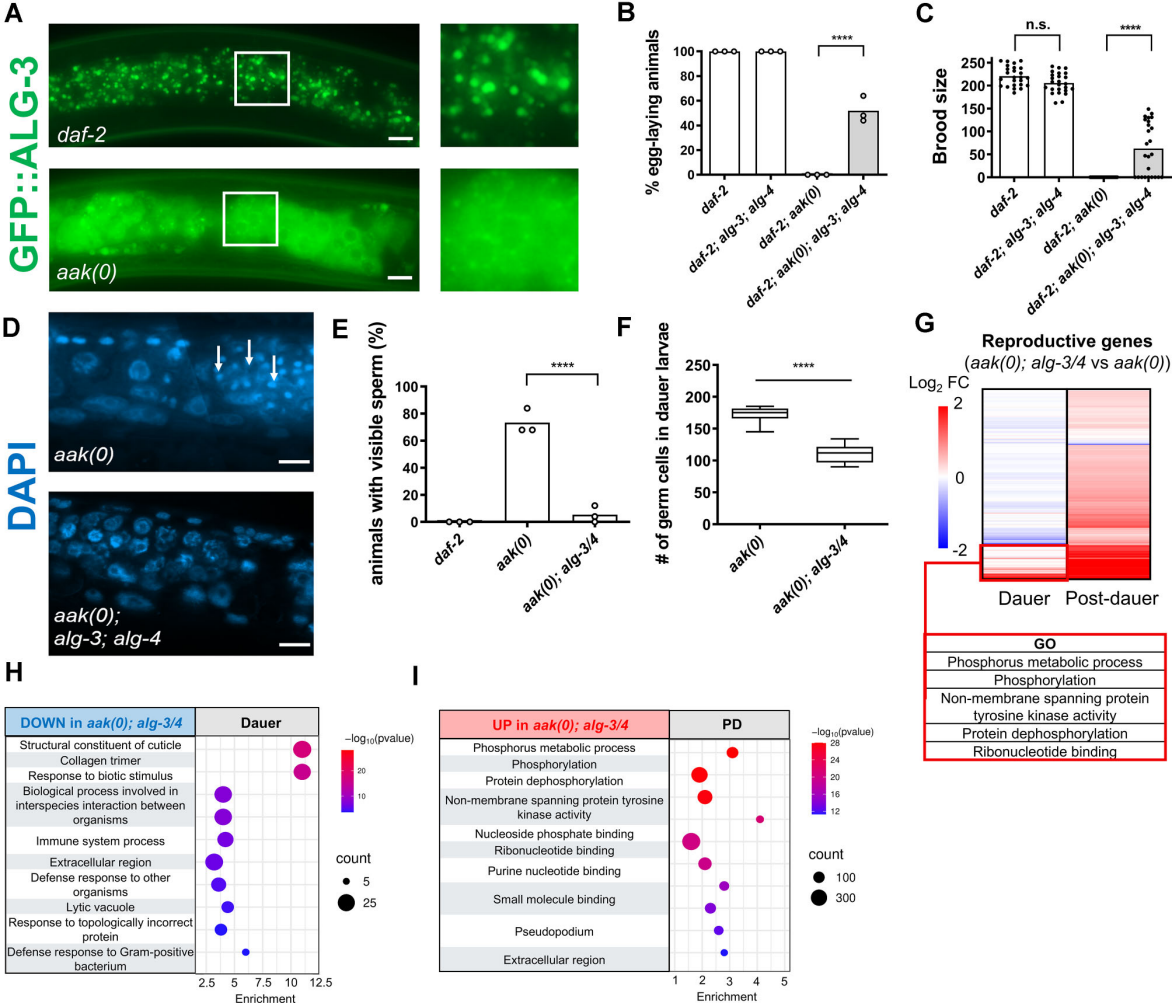
Removing ALG-3/4 ameliorates reproductive defects in AMPK mutants. (**A**) Representative confocal micrographs of GFP::ALG-3 expression in the germ line of Control (top) and *aak(0)* (bottom) dauer larvae after 96 h of growth. All animals are in the *daf-2* background. Arrows indicate GFP::ALG-3 signal. Scale bar = 10 µm. (**B, C**) Post-dauer fertility and brood size of *daf-2, daf-2; alg-3/4, daf-2;aak(0)*, and *daf-2; aak(0); alg-3/4* mutants. Animals were grown at 25°C from synchronized L1s for 96 h before being singled onto plates. After ∼1 week, fertility of each animal was assessed and the total percentage (%) of egg-laying animals per sample was recorded. For brood size assays, animals were singled and the brood size of each individual was measured. Post-dauer fertility data represent three independent trials, with the mean represented by columns and values for individual trials indicated by small circles. *n* = 50 for each trial of post-dauer fertility, *n* = 25 for brood size assays. ****P <0.0001, using one-way ANOVA for the indicated comparisons. (**D**) Representative confocal micrographs of DAPI stained animals depicting the dauer germ line of *aak(0)* (top) or *aak(0); alg-3/4* (bottom) double mutant dauer larvae after 96 h of growth. DAPI staining in dauer was carried out as described in the ‘Materials and methods’ section. All animals are in the *daf-2* background. Arrows in top image indicate spermatids. Scale bar = 10 µm. (**E**) Quantification of animals with visible sperm during the dauer stage following DAPI staining in the indicated mutant strains. The data represent three independent trials, with the mean shown as columns and values for individual trials indicated by small circles. *n* = 50 animals for each trial. ****P <0.0001, ***P <0.001 using one-way ANOVA for the indicated comparisons. (**F**) Quantification of germ cells of *aak(0)* and *aak(0); alg-3/4* mutant dauer animals as detected by DAPI staining. *n* = 25 animals for each trial. ****P <0.0001 using two-tailed *t*-test. (**G**) Top: Heatmap of reproductive gene expression in *aak(0); alg-3/4* versus*aak(0)* dauer and post-dauer (PD) animals. Data represent the comparison of the log_2_ fold change between *aak(0); alg-3/4* versus *aak(0)* samples. Bottom: GO enrichment of upregulated genes in *aak(0); alg-3/4* dauer animals compared to *aak(0)*. (**H, I**) Bubble plots depicting the most enriched GO terms in the *aak(0); alg-3/4* versus *aak(0)* DEG set, ranked by significance based on *P*-value. Left graph depicts genes reduced in *aak(0); alg-3/4* dauer, right graph depicts genes increased in *aak(0); alg-3/4* PD. Size of bubbles indicates the number of genes in their respective categories that were enriched in the indicated dataset. GO enrichment was conducted using the Wormbase Gene Set Enrichment Analysis using q-value threshold of 0.1 [60, 61]. All animals were assessed in a *daf-2* genetic background.

To first validate the observed increases in Argonaute gene expression, we used a GFP::ALG-3 translational fusion reporter and monitored GFP levels in control *daf-2* dauer larvae and in *aak(0)* mutants. High levels of the GFP::ALG-3 reporter were observed in the *aak(0)* dauer larvae, while no GFP expression could be detected in the *daf-2* controls (Fig. 4A). Curiously, the GFP::ALG-3 fluorescence in *aak(0)* dauer larvae was comparable to that of L4 hermaphrodites in wild-type animals [46], although there was less diffuse perinuclear GFP expression in the *aak(0)* dauer larvae compared to L4 animals. Our transgene expression data therefore corroborated our findings that the expression of these Argonautes is premature and inappropriately high in the AMPK mutant dauer larvae.

We crossed an established *alg-3; alg-4* double mutant into our *daf-2* and *aak(0)* mutant strains to determine whether removing these Argonautes might ameliorate the AMPK-dependent reproductive phenotypes. The *alg-3/4* double mutant has been reported to be sterile when grown at 25°C [46]. Since we induce dauer formation by switching *daf-2* animals to 25°C, we were concerned that the reproductive development of our mutants could be affected by this temperature shift. Therefore, to precisely determine the temporal window of the ALG-3/4 temperature sensitivity, we grew *alg-3/4* double mutant animals (without *daf-2*) during specific larval stages at the restrictive temperature (25°C) before shifting them back to 15°C for reproductive development. We noted that *alg-3/4* mutant animals grown from the L1 to L3 stage at the restrictive temperature were nevertheless fertile, so long as they were grown at 15°C during the L4 stage onwards (Supplementary Fig. S4A and B). However, animals that were grown at 25°C exclusively during the L4 stage were sterile. We thus conclude that the L4 stage is the critical temperature-sensitive period for this mutation, and that our method of growing these animals at elevated temperatures before that stage (i.e. during dauer) should have no impact on their fertility.

We tested if the loss of *alg-3/4* could suppress the sterility of post-dauer AMPK mutants. In our *daf-2; aak(0); alg-3/4* mutants (hereafter referred to as *aak(0); alg-3/4)*, we noted that post-dauer animals were significantly more fertile, consistent with a partial suppression that occurs by disabling these two Argonaute proteins, although the brood size of the fertile animals remained lower than the wild type (Fig. 4B and C). This would also imply a reduced population or function of their associated 26G RNAs, which in turn ameliorates reproductive development in AMPK mutants. Given that animals were not fully fertile, it is likely that other small RNA pathways may also be influencing the reproductive development of the AMPK mutant animals, such that the suppression of ALG-3/4 or their associated 26G RNAs is not sufficient to correct all the observed reproductive defects.

Notably, we did not detect premature sperm formation in the AMPK mutant dauer larvae that lacked *alg-3/4* (Fig. 4D-E), suggesting that the premature spermatogenesis that occurs in AMPK mutants arises from the misregulated function of ALG-3/4 and/or their associated 26G RNAs. This is consistent with the established role of ALG-3/4 26G RNAs as being spermatogenesis-specific, as well as findings that ALG-3/4 are required specifically for the proper development and health of sperm in both males and hermaphrodites [46, 75]. We did note a decrease in germ cell numbers compared to *aak(0)* (Fig. 4F), suggesting ALG-3/4 activity must also contribute to the germline hyperplasia typical of AMPK mutants.

We carried out crosses using the sperm-less *fog-2* strain to determine whether sperm health was rescued in the *aak(0); alg-3/4* mutants. Crossing post-dauer *aak(0)* males with *fog-2* females results in partial fertility, but this is greatly increased when crossing *fog-2* females with *aak(0); alg-3/4* males (Supplementary Fig. S4C). Crossing *fog-2* males with post-dauer *aak(0)* or *aak(0); alg-3/4* hermaphrodites resulted in a partial suppression of sterility in the *alg-3/4* animals, but their levels of fertility were nevertheless reduced compared to hermaphrodites that self-mated (Fig. 4B and C), or with *alg-3/4* males crossed with *fog-2* females. In each case, we confirmed that mating occurred by confirming the presence of male progeny. These data allowed us to conclude that the loss of *alg-3/4* corrects sperm development defects that occur in AMPK-deficient dauer/post-dauer larvae, although some oogenic defects may also be partially suppressed.

Given that the loss of *alg-3/4* ameliorates many of the defects associated with *aak(0)* mutants, we wondered whether this was reflected in the gene expression changes as well. We carried out mRNA-Sequencing with the *aak(0); alg-3/4* mutants in the dauer and post-dauer stages. Following differential gene expression analysis, we saw that there was a decrease in some reproductive gene expression in the *aak(0); alg-3/4* mutants compared to *aak(0)* in the dauer stage (Fig. 4G, Top). We also observed an increase in the expression of some genes, and these are enriched for GO terms including phosphorylation and ribonucleotide binding (Fig. 4G, Bottom). This pattern is similar to that of ALG-3/4 gene targets that are impacted by the loss of AMPK (Supplementary Fig. S2E), although only 41 out of 215 genes appear to overlap. ALG-3/4 can suppress gene expression of its targets, and so downregulation of this subset of genes may be one contributing factor to the sterility seen in AMPK mutants. More dramatically, we saw a general increase in post-dauer reproductive gene expression in the *aak(0); alg-3/4* mutants, consistent with the ability of these mutants to rescue fertility. The loss of *alg-3/4* thus partially corrects several of the reproductive defects in *aak(0)* mutants through changes in gene expression, although the transcriptome is not fully restored to its wild-type state.

To our surprise, we did not see a strong enrichment of reproductive genes that were decreased in the *aak(0); alg-3/4* dauer dataset (Fig. 4H). However, when we look at the post-dauer transcriptome (Fig. 4I), the GO enrichment of genes with increased expression strongly resembled that of the gene set decreased in *aak(0)* post-dauer compared to *daf-2* (Fig. 2A, bottom right), suggesting many of the post-dauer changes are reversed in the *alg-3/4* mutant background. Thus, we conclude that the reduction of *alg-3/4* partially suppresses the premature reproductive gene expression present in *aak(0)* dauer larvae (Fig. 4G), which in turn influences the post-dauer transcriptome in favour of reproductive development. The overexpression of reproductive and spermatogenic genes persists, but this may be due to the influence of other small RNA pathways, such as CSR-1 22G siRNAs, which also influence spermatogenic gene expression [79].

Recently, it was revealed that MUT-16 dependent RNAs regulate the timing and expression of ALG-3/4 [80], and we wondered whether this protein might contribute to the misregulated gene expression we observe *aak(0)* dauer larvae. Although this is unlikely, given that MUT-16 loss of function caused defects in ALG-3/4 expression, whereas in *aak(0)* dauer larvae, levels of *mut-16* are increased (Fig. 3A), we nevertheless performed RNAi against *mut-16* in *daf-2, aak(0)*, and *aak(0); alg-3/4* animals and saw no observable changes in ALG-3 expression in *aak(0)* dauer larvae (Supplementary Fig. S4D). Furthermore, we observed no changes in post-dauer sterility or brood size (Supplementary Fig. S4E and F). We validated the efficacy of our RNAi by checking for fold change decreases of *mut-16* in the RNAi treated worms, and saw a decrease in both *aak(0)* and *aak(0); alg-3/4* mutants (Supplementary Fig. S4G). We additionally saw that these RNAi-treated animals were temperature-sensitive sterile, such that growth at 25°C rendered them infertile (Supplementary Fig. S4H andI). This confirmed our *mut-16* RNAi treatment worked as intended. Therefore, the activity of MUT-16 or MUT-16-dependent RNAs do not play a significant role in the misregulation of ALG-3/4 or 26G spermatogenic RNAs in *aak(0)* dauer animals, and that there exists a distinct mechanism by which the absence of AMPK impacts ALG-3/4 and other Argonautes in the dauer stage.

### CSR-1 misregulation contributes to fertility defects in AMPK mutant dauer larvae

Although ALG-3/4 appeared to be the most upregulated Argonautes in AMPK mutant dauer larvae, we noted that levels of most other small RNA pathway components were also increased. The role of small RNA regulators during the dauer stage has not been extensively examined, with the exception of a nuclear Argonaute protein called CSR-1 that acts as a key regulator of gene expression in the germ line and was found to influence multiple aspects of dauer development, including dauer-specific gene expression [81]. CSR-1 regulates a subset of ‘seesaw’ genes that have altered expression in post-dauer adults depending on which environmental trigger resulted in dauer formation.

**Figure 5.**
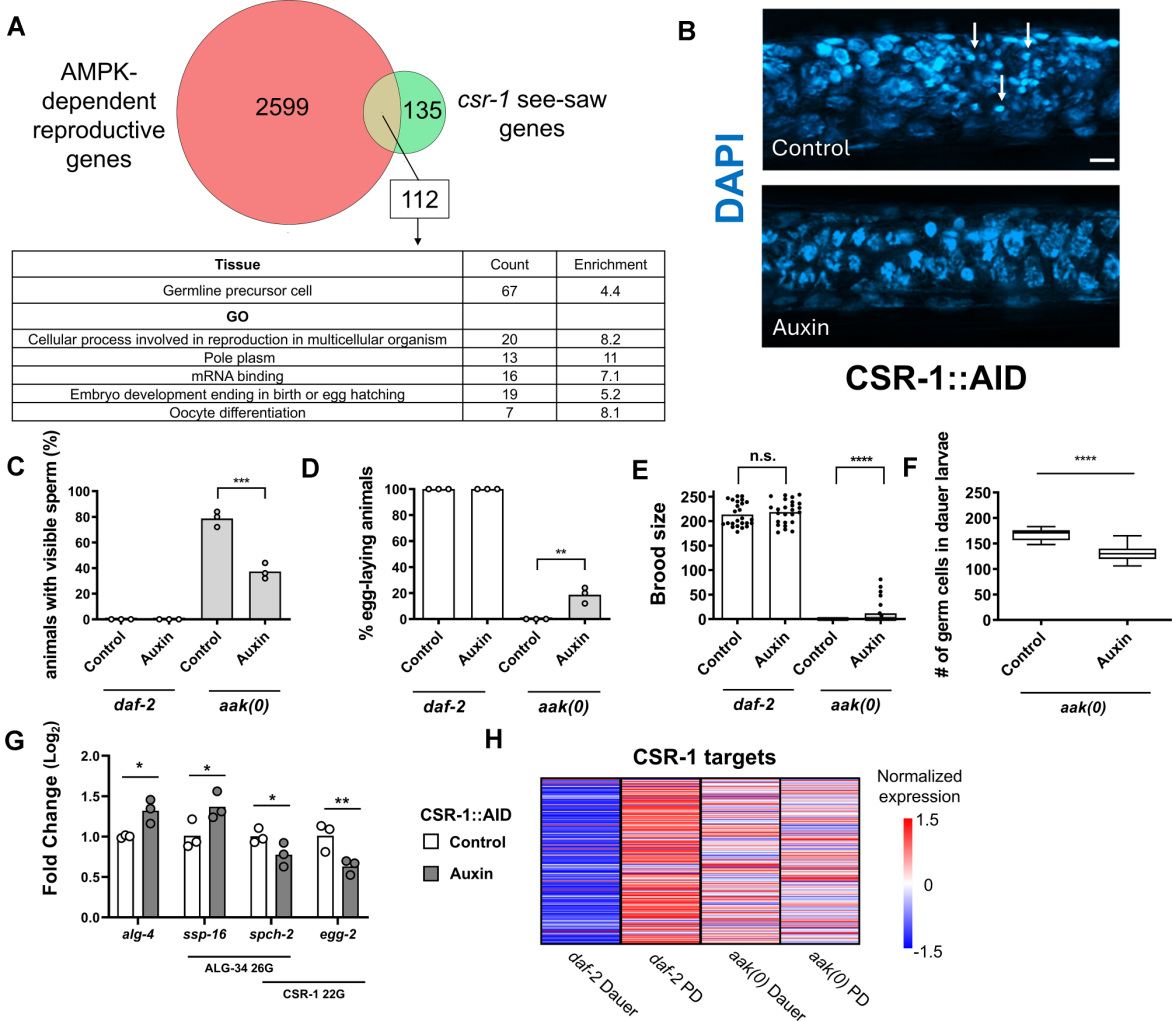
Compromise of CSR-1 during dauer partially suppresses premature spermatogenesis and post-dauer fertility in AMPK mutant dauer larvae. (**A**) Top: Venn diagram comparing AMPK-dependent reproductive genes from Fig. 2B with ‘seesaw’ genes, specifically those enriched in the “WTPhe up::WTStv down” subset from Ow *et al*. (2018) [81], most of which are targets of CSR-1. Bottom: Tissue and GO enrichment of 101 overlapping genes. GO enrichment was conducted using the Wormbase Gene Set Enrichment Analysis using a q-value threshold of 0.1 [60, 61]. “Count” indicates number of genes out of 101 matching respective term. See Supplementary Table S3 for list of genes. (**B**) Representative confocal micrographs depicting DAPI staining of control (top) and auxin-treated (bottom) CSR-1::AID animals during dauer in the *aak(0)* background. Arrows indicate visible sperm. Animals were grown on standard NGM plates and only placed on auxin plates shortly before dauer entry, with DAPI staining performed after 48 h in dauer. DAPI staining in dauer was carried out as described in the ‘Materials and methods’ section. See Supplementary Fig. S5B for protocol details. (**C**) Quantification of CSR-1::AID animals with visible sperm, with and without auxin treatment during the dauer stage, based on microscopy of DAPI-stained animals. The data represent three independent trials, with the means shown as columns and values for individual trials indicated by small circles. *n* = 50 animals for each trial. ****P <0.0001, ***P <0.001 using one-way ANOVA for the indicated comparisons. (**D–F**) Post-dauer fertility, brood size, and germ cell count of *daf-2* and *aak(0)* CSR-1::AID animals with and without auxin treatment during the dauer stage. Animals were grown at 25°C from synchronized L1s for 46 h before being moved to regular NGM or auxin-supplemented plates, then after 48 h, animals were singled onto NGM plates. After ∼1 week, fertility of each animal was assessed and the total % of egg-laying animals per sample was recorded. For brood size assays, animals were singled and brood size of each individual animal was measured. Post-dauer fertility data represent three independent trials, with the mean represented by columns and values for individual trials indicated by small circles. *n* = 50 for each trial of post-dauer fertility, *n* = 25 for brood size assays. ****P <0.0001, **P <0.01 using one-way ANOVA for the indicated comparisons. *n* = 25 animals for germ cell counts, ****P <0.0001 using two-tailed *t*-test. (**G**) Gene expression of indicated genes by RT-qPCR in CSR-1::AID *aak(0)* animals with and without auxin treatment during the dauer stage. Primers targeting *alg-4, ssp-16, spch-2*, or *egg-2* were used. *tba-2* was used as a representative housekeeping gene for comparison. Total RNA was collected after 48 h in dauer. Data from auxin-treated samples were compared to respective control samples for statistical tests. Bottom labels indicate which small RNA family regulates expression of respective genes. Three independent trials were performed for all gene expression assays. *P <0.05, **P <0.001 using two-way ANOVA. Units are log_2_ fold change. (**H**) Heatmap of the expression of gene targets of CSR-1 22G siRNAs, selected based on small RNAs that were enriched in *aak(0)* dauer larvae compared to *daf-2* controls. Data represent normalized transcripts per million values for each gene, with higher relative expression in red and lower in blue. See Supplemental Raw Data for list of genes used in analysis. All animals assessed were in a *daf-2* genetic background and contained a CSR-1::AID tag.

We compared our ∼2000 AMPK-dependent reproductive genes (Fig. 2C) to the 245 genes upregulated in pheromone-induced dauer larvae, but which were downregulated in starvation-induced dauer (referred to as ‘WT_Phe_ up::WT_Stv_ down’ by Ow *et al*.) [81]. We found that there was a significant overlap of 112 genes i.e. roughly 45% of the seesaw genes were also present in our AMPK-dependent subset (Fig. 5A, top; listed in Supplementary Table S3). Conversely, comparing our AMPK-dependent genes to the other set of seesaw genes (WT_Phe_ down::WT_Stv_ up), we noted that only seven genes overlapped.

Ow *et al*. revealed that 95% of the WT_Phe_ up::WT_Stv_ down genes were targeted by the germline Argonaute CSR-1 [42], and further found that CSR-1 function was necessary for the regulation of these seesaw genes during dauer. Given that a significant number of these seesaw genes are also misregulated in *aak(0)* mutants, we suspect the CSR-1 pathway also contributes to the observed changes in gene expression in AMPK-deficient dauer and post-dauer animals, and other small RNA pathways may serve a function as well. Enrichment analysis of the 112 overlapping genes from the two datasets revealed that more than half (67 out of 112) corresponded to genes present in germline precursor cells, and based on GO enrichment, were associated with reproductive and developmental functions (Fig. 5A, bottom). This would be consistent with the small RNA pathways acting as key regulators downstream of AMPK to control reproductive development during periods of starvation or other environmental stress.

We used a CSR-1::GFP reporter strain to monitor levels of the protein, and noted that the protein is strongly visible in *daf-2* dauer larvae in the germ line, and that expression is even more widespread in *aak(0)* dauers, presumably because of the germline hyperplasia present in these animals (Supplementary Fig. S5A). Given its prominent roles in reproductive development and its effects on dauer gene expression, we wondered whether the aberrant levels of CSR-1 in *aak(0)* dauer animals were contributing to the reproductive defects that we observe in these mutants. Because CSR-1 null mutants are sterile [45], we opted to use the auxin-inducible degron system [82] to eliminate CSR-1 in the germ line exclusively during the dauer stage (protocol in Supplementary Fig. S5B), and subsequently used the CSR-1::AID expressing animals to dissect the role of CSR-1 in our mutants.

We performed DAPI staining on CSR-1::AID *daf-2* and *aak(0)* animals, either grown on standard NGM plates (‘Control’) or on auxin-supplemented plates to deplete CSR-1 (‘auxin’). We saw that there was a partial reduction of the premature spermatogenesis phenotype that we see in *aak(0)* dauer larvae (Fig. 5B and C). The number of animals with visible sperm was nevertheless higher than in *aak(0); alg-3/4* mutants, suggesting CSR-1 is only partially contributing to the observed premature spermatogenesis, consistent with previous studies [79]. Furthermore, we saw a slight suppression of post-dauer sterility and germline hyperplasia in the CSR-1 depleted animals (Fig. 5D–F). These data suggest CSR-1, and presumably its target 22G RNAs, influence the germ line of AMPK mutant dauer larvae, but the impact is not as significant as that of ALG-3/4.

We wondered whether CSR-1 was instead affecting the levels of other Argonautes and contributing to the overall increase in small RNA activity. The known targets of different small RNA pathways include Argonautes themselves, suggesting the possibility of cross-regulation, and feedback loops that connect the 22G and 26G RNA pathways have been previously identified [83]. It is possible that CSR-1 promotes the expression of ALG-3/4 and potentially other small RNA pathway factors. We performed RT-qPCR on control and auxin-treated CSR-1::AID dauer larvae in order to assess whether levels of spermatogenic genes were under the control of CSR-1 in *aak(0)* animals. We saw that levels of *alg-4* as well as *ssp-16*, a gene targeted specifically by 26G RNAs, was not decreased in CSR-1 depleted animals, but actually appeared to increase slightly (Fig. 5G). By contrast, *egg-2*, a known target of CSR-1 22G RNAs, did show reduced expression, as expected. Levels of *spch-2*, a spermatogenic gene known to be regulated by both 26G and 22G RNAs, was slightly decreased. Taken together, these data suggest that CSR-1 regulates its oogenic targets like *egg-2* in *aak(0)* dauer larvae, but is not the primary factor contributing to the highly elevated expression and activity of ALG-3/4 and 26G RNAs, and that there must be an alternative mechanism that would explain the misregulation of this pathway.

We cannot yet explain why the levels of *alg-4* and *ssp-16* appear to increase slightly in the absence of CSR-1, but it may be due to reduced 22G levels impacting the dynamics of small RNA populations and influencing the ERI-Dicer complex to favor different small RNA substrates. Indeed, altering the equilibrium levels of specific small RNAs can influence the expression and function of various RNAi pathway components [83]. If the absence of AMPK somehow leads to the significant increase in siRNA levels, these RNAs could themselves promote the expression, stability, and/or function of the Argonaute/RNA complexes [84].

To get a clearer view of how the CSR-1-associated 22G RNAs may be impacting the transcriptome, we looked at the gene expression of known CSR-1 22G targets [42]. We observed that many targets had increased expression in the *aak(0)* dauer stage compared to *daf-2* (Fig. 5H), with expression decreasing in the post-dauer. This pattern of expression is similar to that of ALG-3/4 26G targets (Fig. 3D), although the effects appear to be less dramatic. Finally, we compared the fold change of CSR-1 22G RNAs in *aak(0)* versus *daf-2* dauer to the fold change of their cognate genes (Supplementary Fig. S5C). Unlike the ALG-3/4 26G comparison (Supplementary Fig. S4D), we saw that there was a weaker correlation between the two datasets, and many gene targets appear to have neutral or minimal changes in expression. Nevertheless, we do see several gene targets with elevated levels in *aak(0)* dauer compared to the wild-type, consistent with prior findings that CSR-1 22G RNAs are able to promote expression of their target genes [42]. These data demonstrate the complexity of the relationships that exist between the small RNA pathways and their physiological impact, particularly when they are misregulated to such a degree as we have documented in the *aak(0)* dauer stage.

### Misregulation of small RNA biogenesis contributes to premature spermatogenesis and sterility in AMPK mutants

**Figure 6.**
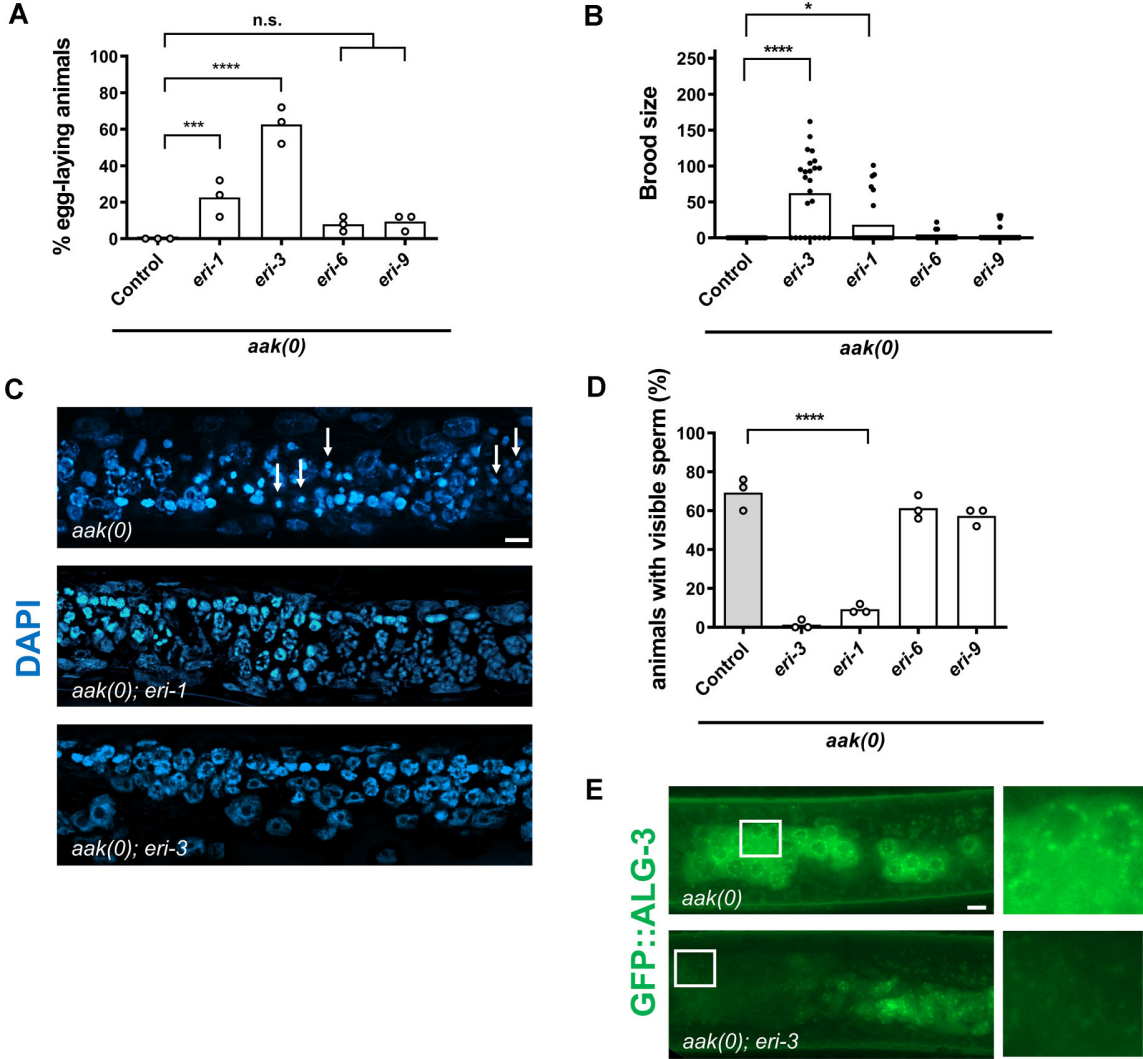
Compromising *eri* gene function suppresses the aberrant ALG-3 expression and premature spermatogenesis in AMPK dauer larvae. (**A, B**) Post-dauer fertility and brood size of *aak(0)* and *eri* double mutants. Animals were grown at 25°C from synchronized L1s for 96 h before being singled onto plates. After ∼1 week, fertility of each animal was assessed and the total % of egg-laying animals per sample was recorded. For brood size assays, animals were singled and brood size of each individual animal was measured. Post-dauer fertility data represent three independent trials, with the mean represented by columns and values for individual trials indicated by small circles. *n* = 50 for each trial of post-dauer fertility, *n* = 25 for brood size assays. ****P <0.0001, ***P <0.001, *P <0.05 using one-way ANOVA for the indicated comparisons. (**C**) Representative confocal micrographs of DAPI staining depicting the dauer germ line of *aak(0)* (top), *aak(0); eri-1* (middle), or *aak(0); eri-3* (Bottom) mutants. Arrows indicate the presence of sperm. DAPI staining in dauer was carried out as described in the methods section). Scale bar = 10 µm. (**D**) Quantification of animals with visible sperm in the indicated genetic backgrounds, based on microscopy of DAPI-stained animals. The data represent three independent trials, with the mean shown as columns and values for individual trials indicated by small circles. *n* = 50 animals for each trial. *P* <0.0001, ***P <0.001 using one-way ANOVA for the indicated comparisons. (**E**) Representative confocal micrographs of GFP::ALG-3 expression in *aak(0)* (top) and *aak(0); eri-3* (bottom) dauer larvae germ line after 96 h of growth. Scale bar = 10 µm. All animals were assessed in a *daf-2* genetic background.

Since the reduction of the two spermatogenic Argonaute proteins ALG-3/4 partially suppressed the sterility of the post-dauer AMPK mutants, we reasoned that the increased expression of both small RNAs and a number of their regulators might also impinge on the timing of sperm production in the *aak(0)* mutant dauer larvae. By altering the levels of 26G RNAs and their regulators, we wanted to determine if these changes could also ameliorate the sterility observed in post-dauer AMPK mutants. Because of their pivotal role in the production of ALG-3/4 26G RNAs, we focused on the *eri* genes, which according to our RNA-Seq analysis also showed an increased expression in *aak(0)* dauer larvae (Fig. 3A). We found that *daf-2; aak(0); eri-1* (hereafter *aak(0); eri-1*) and *daf-2; aak(0); eri-3* (hereafter *aak(0); eri-3)* mutants exhibited a suppression of post-dauer sterility seen in AMPK mutants (Fig. 6A and B), but this was not seen with mutants of *eri-6* or *eri-9*, suggesting that the high levels of small RNAs contribute significantly to AMPK mutant post-dauer sterility. Both ERI-1 and ERI-3 form a complex with Dicer and are required for the accumulation of many types of siRNAs [85, 86], whereas ERI-6/9 are primarily required for ERGO-1 class 26G RNAs, and their absence leads to a reduction in ERGO-1 class 26G RNAs [87, 88]. This may explain why the compromise of *eri-6* or *eri-9* do not impact the fertility of these animals to the same degree, since a smaller subset of RNAs are affected by their absence.

Many 26G RNA target genes have been linked to spermatogenesis [44]. Therefore, the premature spermatogenesis that we observed in AMPK mutant animals could equally be a consequence of misregulated 26G spermatogenic RNAs. Spermatids are very distinct following DAPI staining due to the densely compacted DNA typical of their nuclei (Supplementary Fig. S1A). In dauer larvae that lack all AMPK signaling, the sperm nuclei are clearly discernible in the mutant gonads and are no longer present in animals that lack *eri-1* or *eri-3*. Based on our DAPI staining, we noted that ∼10% of *aak(0); eri-1* mutants exhibited premature dauer spermatogenesis, compared to ∼60% in *aak(0)* mutants, while almost no *aak(0); eri-3* mutant animals demonstrated untimely sperm formation (Fig. 6C and D). These findings suggest that compromise of a subset of factors involved in small RNA biogenesis can ameliorate many of the germline-related phenotypes associated with the loss of AMPK function, largely through their effects on the regulation and timing of spermatogenesis.

We noted that levels of ALG-3 were high during the dauer stage in *aak(0)* mutants, and this was consistent with our RNA-Seq data where *alg-3* and *alg-4* transcripts are six- to nine-fold higher than controls (Fig. 3A). Notably, we observed greatly reduced levels of GFP::ALG-3 in dauer larvae of *aak(0); eri-3* mutants compared to *aak(0)* mutants (Fig. 6E). These data imply that in the *aak(0)* dauer larvae, ALG-3 Argonaute expression and/or stability is influenced by the increased levels of this class of small RNAs, rather than the converse, where the increased Argonaute levels might be responsible for the accumulation of specific small RNA populations. If this is true, then AMPK or one of its downstream targets must modulate small RNA levels, rather than affecting the abundance or the activity of ALG-3/4 *per se*. We also quantified levels of a subset of ALG-3/4 26G RNA target genes using RT-qPCR, and found their levels greatly reduced in both *aak(0); alg-3/4* and *aak(0); eri-3* mutants compared to *aak(0)* animals (Supplementary Fig. S6A), suggesting that the loss of *eri-3* function results in a downregulation of ALG-3/4-dependent target gene expression in the AMPK mutants.

To confirm whether the loss of *eri* gene function can correct small RNA homeostasis in AMPK mutants, we performed RT-qPCR of selected ALG-3/4 26G siRNAs using Taqman [89]. We used established Taqman primers to measure the levels of two ALG-3/4 26G siRNAs that are enriched in *aak(0)* dauer larvae based on our small RNA-Seq data, targeting the loci T27A3.3 and F35E2.5. We compared 26G siRNA expression in *aak(0)* and *aak(0); eri-3* mutants, and for samples we used both dauer larvae as well as adult animals that had not transited through dauer. Adult *eri-3* mutants should have reduced levels of siRNAs, including 26G spermatogenic siRNAs [87] and would thus serve as a positive control for our Taqman assay. Following RT-qPCR with these assays, we found that the levels of the two selected 26G RNAs in both dauer and adult *aak(0); eri-3* mutants were reduced compared to *aak(0)* animals (Supplementary Fig. S6B). Given that ALG-3 expression appears to be responsive to levels of small RNAs, and potentially ALG-3/4 26G spermatogenic RNAs specifically, it is highly likely that the changes in the siRNA populations of *aak(0)* dauer animals result from the absence of AMPK, and induce many of the downstream developmental defects typical of these mutants through their effects on gene expression.

### The increased expression of ALG-3 in AMPK mutant dauer larvae is affected by 26G small RNA levels

The loss of *eri-3*, and presumably the consequent decrease in the levels of many small RNAs, greatly reduced the aberrant expression of ALG-3 in our *aak(0)* dauer animals. While this may be a result of gene expression being indirectly reduced due to the downregulation of small RNAs, our RT-qPCR data suggest that levels of *alg-3* decrease only slightly when in *eri-3-*defective animals (Supplementary Fig. S6A). When we first noticed the stark difference in GFP::ALG-3 levels in the *aak(0); eri-3* mutants (Fig. 6D), we wondered whether reduced levels of small RNAs influenced the activity or expression of ALG-3. Previously, it was demonstrated that the presence of miRNAs can stabilize their cognate unbound or Apo-Argonautes, and that this facilitates their function [84]. This phenomenon may also be affecting ALG-3/4 levels in the *aak(0)* mutant dauer animals.

To test whether ALG-3 levels change in response to RNA levels, we first attempted soaking dauer larvae with RNA extracted from *aak(0)* dauer larvae, which would have high levels of RNAs, including small RNAs (Supplementary Fig. S7A). We have previously shown that this soaking protocol can bring about phenotypic changes in AMPK mutants, suggesting animals can take up these RNAs and alter their gene expression accordingly [68]. Following this treatment, we observed an increase in the overall levels of GFP::ALG-3 in the germ lines of these treated larvae, stronger than control samples, but not as strong as animals without the *eri-3* mutation (Supplementary Fig. S7B and C). However, the expression of GFP::ALG-3 was less discrete than in animals that were not soaked with RNAs. We suspect that the presence of other RNA types in these samples may be a confounding factor, and ideally we wanted to know whether it is spermatogenic 26G RNAs, specifically, that influence ALG-3 expression.

**Figure 7.**
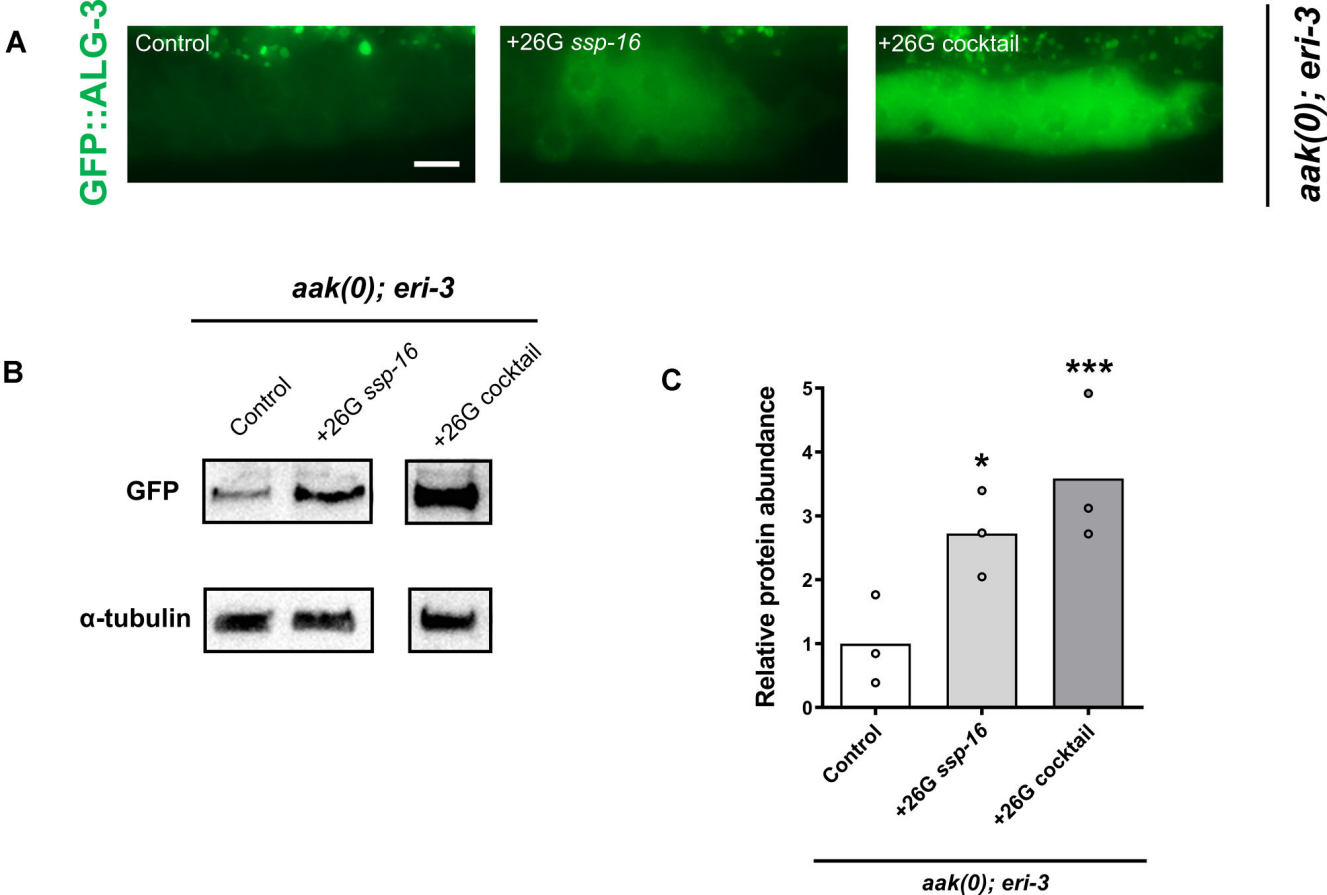
Soaking with 26G RNAs increases ALG-3 expression in AMPK and *eri-3* mutant dauer larvae. (**A**) Representative confocal micrographs of GFP::ALG-3 expression in *aak(0); eri-3* dauer animals soaked with 26G RNAs or a control RNA solution. Animals were grown in liquid at 25°C for 96 h, soaked with either a control solution of TE buffer, an *in vitro* synthesized 26G RNA targeting the spermatogenic gene *ssp-16* dissolved in TE buffer, or a cocktail of three *in vitro* synthesized 26G RNAs targeting *ssp-16, spch-2*, and *msrp-2*. Scale bar = 10 µm. (**B**) Representative western blots performed from *aak(0); eri-3* dauer animals soaked with 26G RNAs or a control RNA solution. Soaked animals were washed and pelleted in PBST, then frozen or used immediately for western analysis. Antibodies against GFP were used to measure levels of GFP::ALG-3, while antibodies against α-tubulin serve as a loading control. (**C**) Quantification of bands from western blots in panel (B). Band intensity was measured for each GFP and α-tubulin band along with nearby background intensity. Adjusted band intensities for GFP were then divided by values for the corresponding α-tubulin band to calculate relative intensity. Finally, the average relative intensity of the Control bands from three biological replicates was calculated, and ‘Relative Protein Abundance’ was calculated by dividing every relative intensity value by that average. Data represents three independent trials. *P <0.05, ***P <0.001 using one-way ANOVA. All animals were assessed in a *daf-2; aak(0); eri-3* genetic background.

We thus generated 26G RNAs by *in vitro* transcription (Supplementary Fig. S7D and E) [67], and we repeated our soaking experiment, first using a solution of a single 26G RNA that targeted the spermatogenic gene *ssp-16*, chosen because its corresponding 26G RNA levels were greatly elevated based on our small RNA-Seq data. Following our soaking protocol, we observed an increase in the levels of GFP::ALG-3 (Fig. 7A), although the effect was inconsistent, whereby only some worms displayed an increase in GFP. We next tested a cocktail combining three different 26G RNAs that targeted *ssp-16, spch-2*, and *msrp-2*. Soaking with this solution led to a very strong increase in GFP::ALG-3 levels in the germ line (Fig. 7A, right). Furthermore, we quantified the GFP levels using western blot and saw that soaking with the *ssp-16* 26G solution led to a moderate increase in ALG-3 levels, but soaking with the cocktail resulted in very strong enhancement of GFP::ALG-3 expression (Fig. 7B and C).

We thus conclude that the levels of ALG-3 in our *aak(0); eri-3* mutants are responsive to 26G RNA abundance, and that the reduced small RNA levels resulting from absence of *eri-3* likely explains the diminished ALG-3 levels in this strain, potentially due to the instability of the unbound Apo-Argonaute. Consistent with this, when we add back a population of 26G RNAs by soaking, the ALG-3 levels are stabilized. The dynamics of small RNA populations are therefore crucial for proper timing of reproductive development, and when these populations are misregulated, as is the case in AMPK-deficient dauer larvae, this contributes to aberrant stabilization/activation of the Argonautes.

Through RNA-Seq analysis of dauer and post-dauer AMPK-deficient animals, we have identified sweeping transcriptomic changes that result from the absence of AMPK, specifically highlighting the premature onset of reproductive development based on changes in gene expression. Furthermore, we note that the significant increase in ALG-3/4 in *aak(0)* dauer larvae correlates strongly with our RNA-Seq data and the physiological changes that manifest in the absence of this protein kinase, namely ALG-3/4-dependent premature spermatogenesis. Finally, we reveal that disabling RNA biogenesis not only corrects several *aak(0)* dauer defects, but also affects the levels of ALG-3 itself, suggesting that the lack of AMPK may not directly impinge on the Argonautes *per se*, but instead increases small RNA biogenesis or alters the balanced proportions of the various small RNA classes. This results in an increase in the steady state abundance of 26G RNAs allowing them to feed back and potentially stabilize ALG-3.

## Discussion

The induction of developmental pathways, particularly reproductive development, is exquisitely timed in most organisms, but it is also tied to resource availability and energy homeostasis. A lack of key resources can lead to reproductive defects or result in animals simply foregoing reproductive development until growth conditions improve [4, 6]. This reproductive trade-off is a common strategy shared among several animal groups, while the mechanism that permits this plasticity has not been characterized generally, nor is it clear that any one mechanism is conserved. Nevertheless, organisms continuously monitor resource availability to ensure development does not proceed in sub-optimal conditions, thereby decreasing their reproductive fitness. This highlights the importance of conserved metabolic regulators, such as AMPK, which tie cellular energy status to development.

Our laboratory and others have highlighted the role of AMPK as a lynchpin in the correct regulation of development during periods of duress [21, 33, 90, 91]. Curiously, many of the reproductive defects that have been identified in *C. elegans* are associated with disruptions to small RNA homeostasis [34]. We sought to further examine this relationship and understand how changes in small RNA levels can impact reproductive development in such a dramatic manner, and how the abundance or function of the various small RNA pathways are affected when AMPK is absent.

The role of AMPK in mediating reproductive fitness has been recently examined in mammalian models [26], but less so in *C. elegans*, potentially because loss-of-function AMPK mutants do not significantly affect fertility during optimal conditions. However, this is dramatically different for AMPK mutant animals that transit through the dauer stage [21]. This unique role of AMPK is likely due to the strict energetic requirements associated with the dauer stage, or rather the decision not to enter dauer and instead undergo reproductive development. In its natural setting, *C. elegans* are predominantly found in the dauer state [92], and factors such as AMPK might ensure that the germ line remains quiescent during this period, as continued reproductive development in a suboptimal growth environment can have severe negative consequences on the survival and/or fecundity of both parents and their offspring.

In mammalian models, AMPK restricts cell growth through suppression of the mTOR pathway in conditions during nutrient scarcity [18, 93]. It is therefore not surprising that AMPK similarly serves to restrict development during the dauer stage in *C. elegans*, where the animals must subsist solely on a limited supply of stored nutrients, potentially for several months [94]. Preserving the quiescence of the germ line during periods of energy stress is critical such that, when animals resume development, they can reproduce without issue. AMPK maintains this quiescence, and thus when it is absent, animals exhibit multiple somatic and germline/reproductive defects in the post-dauer stage.

The inability of AMPK-deficient *C. elegans* to adjust to the energy requirements of the dauer stage has consequences beyond simply altering their energy metabolism. We have previously shown that these animals are unable to properly utilize their stored lipid reserves during dauer, leading to greatly reduced survival [28]. Our examination of reproductive defects in *C. elegans* AMPK mutants suggests there are other means by which AMPK ensures dauer quiescence, such as through regulation of small RNA pathways. We characterized numerous defects in the germ line of these mutants, with animals exhibiting premature spermatogenesis and germline hyperplasia in the dauer stage (Supplementary Fig. S1A and B), in addition to highly penetrant post-dauer sterility.

While we previously identified several pathways that regulate the observed AMPK mutant phenotypes [12, 34], it remained unclear how the loss of AMPK was able to induce such widespread physiological and developmental changes, specifically in the dauer stage. Our RNA-Seq analysis of dauer and post-dauer AMPK mutants allowed us to identify sweeping alterations in gene expression that primarily involved genes associated with reproduction and small RNA pathways (Figs 2B–D, and 3A). Similarly, our small RNA-Seq revealed that the vast majority of small RNAs were elevated in *aak(0)* dauer and post-dauer animals (Fig. 3B and C, and Supplementary Fig. S3C). We further demonstrated that many of these gene expression changes can be tied directly to reproductive defects observed in these mutants (Figs 3E and F, and 4B–E). The dauer stage requires significant remodeling on a tissue, gene expression, and metabolic level [95]. The large number of DEGs that we noted in our RNA-Seq data suggests AMPK is a critical mediator of these changes, affirming its role as a master regulator of growth during times of nutrient stress.

The GO enrichment of the RNA-Seq dataset indicates that there is a significant change in the regulation of reproductive genes resulting from the loss of *aak(0)* and passage through dauer (Fig. 2B). One concern we had was that *aak(0)* dauer larvae exhibit germline hyperplasia, and thus possess almost five times more germ cells than their wild-type counterparts [34]. This increased number of germ cells does contribute to the enrichment of reproductive genes in *aak(0)* dauer. However, while we saw a decrease from our initial quantification of gene expression changes after normalizing the changes to a germline marker, *pgl-1*, there were still many reproductive genes with abnormally elevated expression in *aak(0)* dauer larvae (Supplementary Fig. S2E). The sharp increase in spermatogenic gene expression is particularly notable and difficult to explain simply by referring to the increased germ cells (Fig. 2D). Furthermore, these additional germ cells cannot account for the differences in gene expression observed in post-dauer animals, where we noted reproductive gene expression is lower in *aak(0)* mutants compared to wild-type controls (Fig. 2B). Therefore, we conclude that premature reproductive development indeed occurs in *aak(0)* dauer larvae, and the reproductive gene enrichment is most likely not merely a result of altered germ cell numbers. Nevertheless, it is worth noting that that some of our observations regarding the transcriptome may reflect the increase in germ cell abundance.

When considering the specific classes of reproductive genes that are upregulated exclusively in AMPK mutant dauer larvae, but not upregulated in control post-dauer adults, we noted many targets that were enriched in male tissues. Notably, both *alg-3* and *alg-4*, Argonautes associated with spermatogenic 26G RNAs, had the highest level of expression among the subset of small RNA pathway genes (Fig. 3A), and the gene targets of 26G spermatogenic RNAs were also enriched in the AMPK mutant dauer population (Fig. 3D). Transcriptomic analysis of *alg-3/4; aak(0)* mutants revealed a slight decrease in reproductive gene expression levels during dauer, and a robust increase in reproductive gene expression in the post-dauer adult (Fig. 4G). This suggests elevated levels of ALG-3/4 were impacting the levels of reproduction-associated transcripts in the *aak(0)* mutants. Our observation that spermatogenesis occurs prematurely in AMPK dauer larvae is consistent with these findings, providing a clear link between changes in small RNA levels, gene expression, and reproductive development. Conversely, knockout of *alg-3/4* not only eliminated the premature spermatogenesis phenotype (Fig. 4D and E), but also modestly reduced germline hyperplasia (Fig. 4F) and partially restored fertility to AMPK mutant post-dauer animals (Fig. 4B and C), suggesting the spermatogenic defects are significant contributors to the sterility. Given the strong correlation between ALG-3/4 26G RNA levels and the expression of their cognate genes (Supplementary Fig. S3D), there is a clear throughline between the activity of these Argonautes, their associated small RNAs, and reproductive gene expression programs.

Sperm development rarely occurs before the L4 stage, but it can happen in some mutant backgrounds [96], in addition to the AMPK mutants (Supplementary Fig. S1A). Because of the lack of energy resources, AMPK likely reinforces a block to germ cell development and concomitantly spermatogenesis. As a result, its absence underscores a relaxed developmental restriction that occurs during the dauer stage, such that germ cells can differentiate to sperm, an event that would otherwise not occur in the L3 stage. In *lin-23* mutants that lack the β-TrCP F-box protein orthologue, sperm differentiation also occurs prematurely during the L3 indicating that a β-TrCP target that is normally eliminated, is stabilized and allows spermatogenesis to proceed prematurely. Currently, it is unclear whether these pathways are related, but the LIN-23 F-box protein and AMPK might target key players in the ALG-3/-4 pathway that enhance spermatogenesis, such that their loss of function results in a similar outcome. Nevertheless, the role of AMPK in regulating spermatogenesis and its contribution to sperm function has been highlighted in numerous species [24, 97], and therefore AMPK might play similar roles in *C. elegans*, as our transcriptomic data indicates.

The widespread effects on gene expression following AMPK compromise is quite surprising. It is remarkable that over 2000 genes, or ∼10% of the predicted genes in the *C. elegans* genome, are dynamically affected by the loss of a single protein kinase (Fig. 2C). It is hard to imagine how this could occur without invoking one or more kinase-responsive transcription factors at the top of a gene expression hierarchy that act both as AMPK targets and subsequent downstream regulators. This large-scale gene regulation could however occur due to the activity of small RNAs and their master regulators, and/or a combination of both upstream transcriptional regulators and small RNA effectors. Based on our RNA-Seq and genetic analyses, the activity of CSR-1 and other Argonautes is higher in AMPK mutant dauers, contributing to changes in gene expression and ultimately inducing some, if not many, of the phenotypic defects we observe. Consistent with this, we noted a clear overlap between the dynamically regulated genes identified in our RNA-Seq analysis and a large number of ‘seesaw’ genes under the control of CSR-1 [81] (Fig. 5A).

CSR-1 itself is very strongly expressed in the germ line of AMPK mutant dauer larvae (Supplementary Fig. S5A), and has established roles in regulating both germline development via small RNAs, and the dauer and post-dauer transcriptome [42, 79, 81]. We thus wondered whether CSR-1 was acting as a major regulator of other small RNA pathways. The removal of CSR-1 only partially rescues the reproductive defects we see in AMPK mutants (Fig. 5C–F), however, and does not appear to reduce the levels of spermatogenic genes (Fig. 5G). Furthermore, the effect of CSR-1 22G RNAs on gene expression appear less dramatic than those of ALG-3/4 (Fig. 5H and Supplementary Fig. S5C). While CSR-1 does influence the germ line of AMPK mutant dauer larvae, we cannot attribute the widespread increase in small RNAs or their cognate Argonautes to the function of this one protein.

On a transcriptomic level, we see an upregulation of nearly every Argonaute in AMPK-deficient dauer larvae, along with their associated factors (Fig. 3A and Supplementary Fig. S3A). Our data, and more recent findings from others, indicate that these changes may reflect increases in the abundance of their associated siRNA. Regulation of Argonaute abundance by small RNAs has been observed before [80]. It remains unclear, however, how or why the absence of AMPK induces these increases in small RNAs and consequently their regulators. One possibility is that the kinase regulates a major upstream modulator of small RNA pathways or biogenesis that must be blocked during the quiescent dauer stage, and this inhibition is compromised when AMPK is absent, leading to a deleterious misregulation of several different classes of small RNAs. The resulting disequilibrium manifests as reproductive defects, including the premature reproductive development that is instructed by the abnormal small RNA signaling. Blocking specific small RNA pathways is therefore critical during periods of quiescence, such as the dauer, and our data suggests that AMPK may be a major regulator of this inhibition given the widespread misregulation that occurs in its absence.

The increase in ALG-3 abundance as a result of small RNAs has recently been demonstrated, where *mut-16-*dependent small RNAs regulate the timing of ALG-3/4 expression to ensure thermotolerant sperm [80]. Our work unveils a similar system whereby AMPK downregulates the activity of ALG-3/4 during the quiescent dauer stage, ostensibly through adjustments to small RNA populations. However, the increased ALG-3/4 expression in AMPK mutant may result from a distinct mechanism. Levels of *mut-16* are elevated in AMPK mutant dauer larvae (Fig. 3A), whereas it is the absence of *mut-16*, and subsequent changes in target small RNA repertoire, that leads to mistiming of ALG-3/4 expression [80]. We note that there are no changes to post-dauer fertility in either *aak(0)* or *aak(0); alg-3/4* animals subjected to *mut-16* RNAi, suggesting MUT-16 does not play a role in the misregulation of 26G RNAs in *aak(0)* dauer animals. (Supplementary Fig. S4C and D). Furthermore, regulation of ALG-3/4 by MUT-16 appears to be important as a heat stress response, whereas the effects on the small RNA pathways mediated by AMPK likely serve as a response to the metabolic stresses of the dauer stage.

We demonstrated that the expression of ALG-3 is sensitive to small RNA levels, specifically to the levels of the target 26G RNAs to which it normally binds. We initially suspected this when we saw a dramatic decrease in GFP::ALG-3 expression in animals lacking *eri* genes, required for the biogenesis of small RNAs (Fig. 6D) and which are upregulated in *aak(0)* dauer animals (Fig. 3A). Our soaking experiments suggested that providing additional 26G RNAs was able to reverse this effect, restoring strong ALG-3 expression in the germ line (Fig. 7, and Supplementary Fig. S7B and C). This may also explain why the levels of other Argonaute proteins are increased to such a dramatic degree in AMPK mutant dauer larvae and maintained throughout the stage (Fig. 4A and Supplementary Fig. S5A). Rather than AMPK or some downstream target regulating the various Argonautes directly, it may be that levels of small RNAs are modulated in order to balance the effects of each RNAi pathway. The Argonautes could be stabilized by the presence of their own target small RNAs, as has been suggested with AGO-1 and microRNAs in *Drosophila* [84]. Future experiments could examine the precise mechanism by which small RNA populations influence their respective Argonautes, and whether the effect we described here with ALG-3 pertains to other Argonautes and their cognate RNA pathways.

We thus propose that AMPK is required for the proper regulation of ALG-3/4, and other small RNA effectors in order to delay reproductive gene expression, particularly sperm development, and preserve quiescence in response to the energetic stress associated with the dauer stage. When AMPK is absent, this regulation is disrupted, resulting in greatly increased siRNA levels. This leads to the upregulation or stabilization of ALG-3/4, and potentially other Argonautes such as CSR-1, bringing about the premature onset of reproductive development. As we showed with our transcriptomic analysis, many of the DEGs in AMPK mutants are CSR-1 specific targets (Fig. 5A and G), and many ALG-3/4 pathway targets are also upregulated (Fig. 3D and Supplementary Fig. S3D), indicating a clear throughline between misregulated small RNA populations and subsequent gene expression changes, all stemming from the absence of a single metabolic kinase, AMPK.

Previously, we showed that small RNAs of somatic origin can be transported to the germline to preserve germline integrity during the dauer stage [34]. Our current study does not provide evidence that 26G or other small RNAs are transported across tissues, although we do see significant impacts in the germ line that stem from the absence of AMPK, including elevated expression of Argonautes such as ALG-3 (Fig. 4A). Further study will be required to determine why small RNA pathways are perturbed so dramatically in the germ line, and if/how upstream small RNA biogenesis factors, including DCR-1, may be regulated by AMPK.

We demonstrate that AMPK is required for the proper timing of reproductive development through its ability to impinge on small RNA abundance and function, specifically in response to nutritional cues. This ensures that animals only execute reproductive development when there are sufficient resources and may thus be considered as a major effector of the reproductive trade off that occurs downstream of resource scarcity. AMPK has conserved roles in regulating development in response to nutrition [33, 98], and here we identify yet another axis through which it carries out this regulation—namely, by modulating populations of small RNAs, which in turn influence the expression and activity of their cognate Argonautes. While the importance of small RNAs in *C. elegans* development has been thoroughly characterized [37, 38, 99], it is notable that they play such a pivotal role during periods of developmental plasticity, such as dauer [100], and are tied intricately to the function of energy-sensing factors such as AMPK.

Further analysis of this regulatory axis may reveal that other Argonautes, and not only ALG-3/4, are responsive to these drastic changes in small RNA populations. It may indeed be possible to induce reproductive development in otherwise quiescent animals by artificially elevating levels of small RNAs, either through genetic manipulation or exogenous addition of RNAs. Our work nevertheless highlights the significant impact of ALG-3/4 26G and other siRNAs on reproductive development, while also underscoring the importance of regulating the timing of small RNA expression. As such, challenging environmental fluctuations can be met with a rapid, adaptive response on gene expression, which is mediated through transcription-dependent and, potentially, transcription-independent mechanisms.

## Supplementary Material

gkag555_Supplemental_Files

## Data Availability

Raw data and gene sets used for figure preparation is provided with this manuscript and can be found in supplementary data file excel sheet. Processed mRNA-Seq and small RNA-Seq data are provided in supplementary excel sheets. Processed mRNA-Seq data can also be accessed through Gene Expression Omnibus, GEO #: GSE316932, with raw files available through Sequence Read Archive using the BioProject ID: PRJNA1244352. Small RNA-Seq can be accessed through GEO #: GSE248834.
