# Supplementary material for: AMPK tunes reproductive gene expression and small RNA homeostasis to mediate timely germ cell development
Source: Nucleic Acids Res. 2026 Jun 8;54(11):gkag555. doi: 10.1093/nar/gkag555 (PMC13291822; doi:10.1093/nar/gkag555)
Supplement: gkag555_Supplemental_Files [file gkag555_Supplemental_Files.zip › Supplementary_Figures_and_Tables.pdf]

SUPPLEMENTARY FIGURES AND TABLES

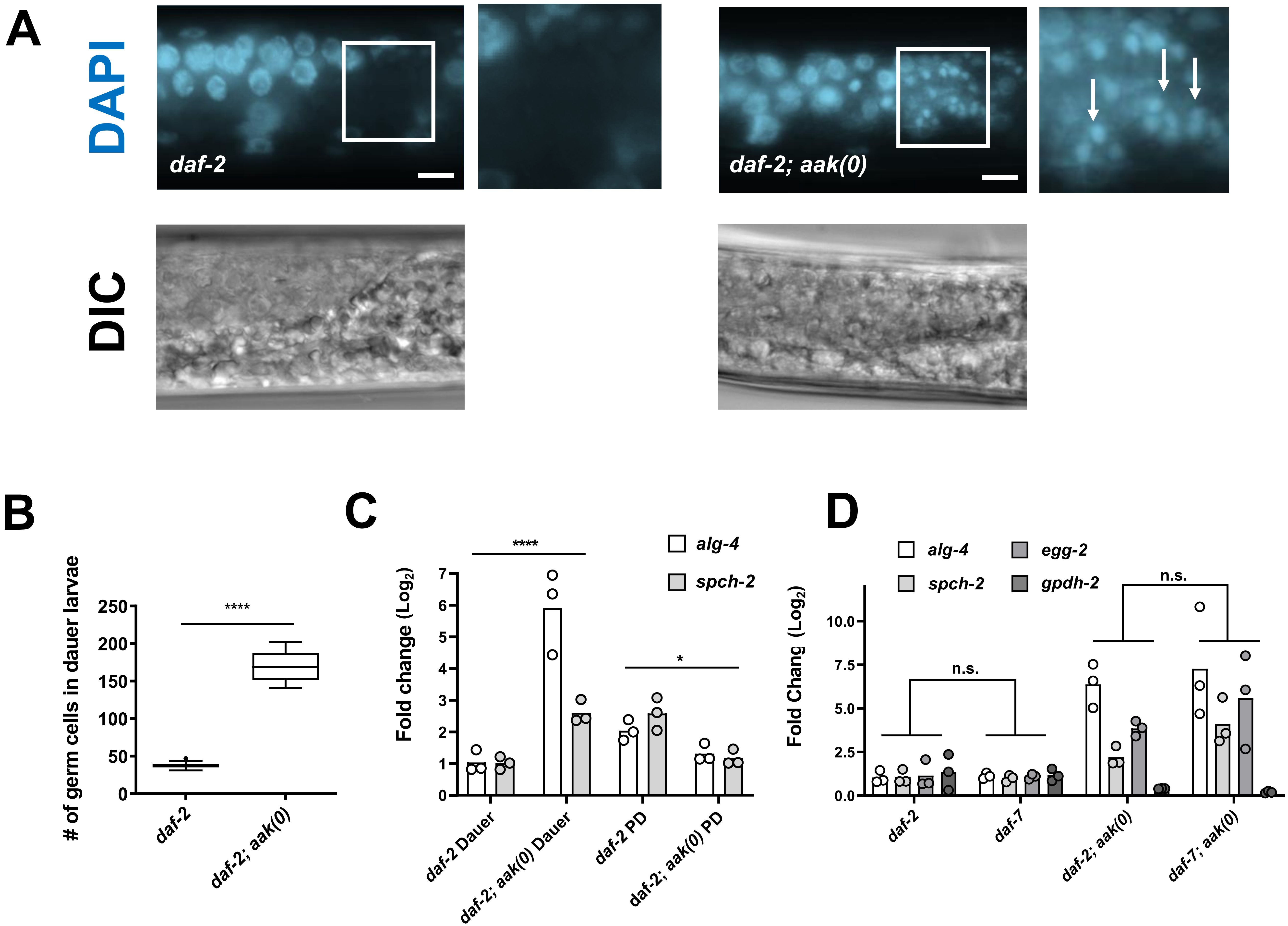

Figure S1. AMPK mutant animals exhibit premature dauer spermatogenesis, dauer germ line hyperplasia, and post-dauer sterility

A) Representative micrographs of DIC and DAPI staining depicting the dauer germ line of *daf-2* (left) and *daf-2; aak(0)* (right) mutants. Arrows indicate the presence of sperm. DAPI staining in dauer was carried out as described in the methods. Scale bar = 10 µm.

B) Quantification of germ cells of *daf-2* and *daf-2; aak(0)* dauer animals as detected by DAPI staining. n = 25 animals for each trial. ****p < 0.0001 using two-tailed t test.

C) Gene expression of *daf-2* and *daf-2; aak(0)* dauer and post-dauer (PD) animals as determined by RT-qPCR using primers for *alg-4* or *spch-2. tba-2* was used as a housekeeping gene for ΔCt calculations, and all data were normalized to *daf-2* dauer values. Statistical comparisons in *daf-2* PD were performed in comparison to equivalent *daf-2* dauer values. Statistical comparisons in *aak(0)* were performed against equivalent *daf-2* data for each gene. Three replicates per group, *p<0.05, ****p < 0.0001 using one-way ANOVA for comparisons. Units are Log_2 fold change.

D) Gene expression of *daf-2, daf-2; aak(0), daf-7* and *daf-7; aak(0)* was performed using primers for *alg-4*, *spch-2, egg-2* or *gpdh-2* in the treatment groups in the dauer stage. *tba-2* was used as a housekeeping gene for ΔCt calculations, and all data were normalized to *daf-2* values. Statistical comparisons were performed against equivalent *daf-2* or *daf-2; aak(0)* datasets for *daf-7* and *daf-7; aak(0)* data, respectively. Each group represents three replicates, **p < 0.01, ***p < 0.001, ****p < 0.0001 using one-way ANOVA for comparisons. Units are Log_2 fold change.

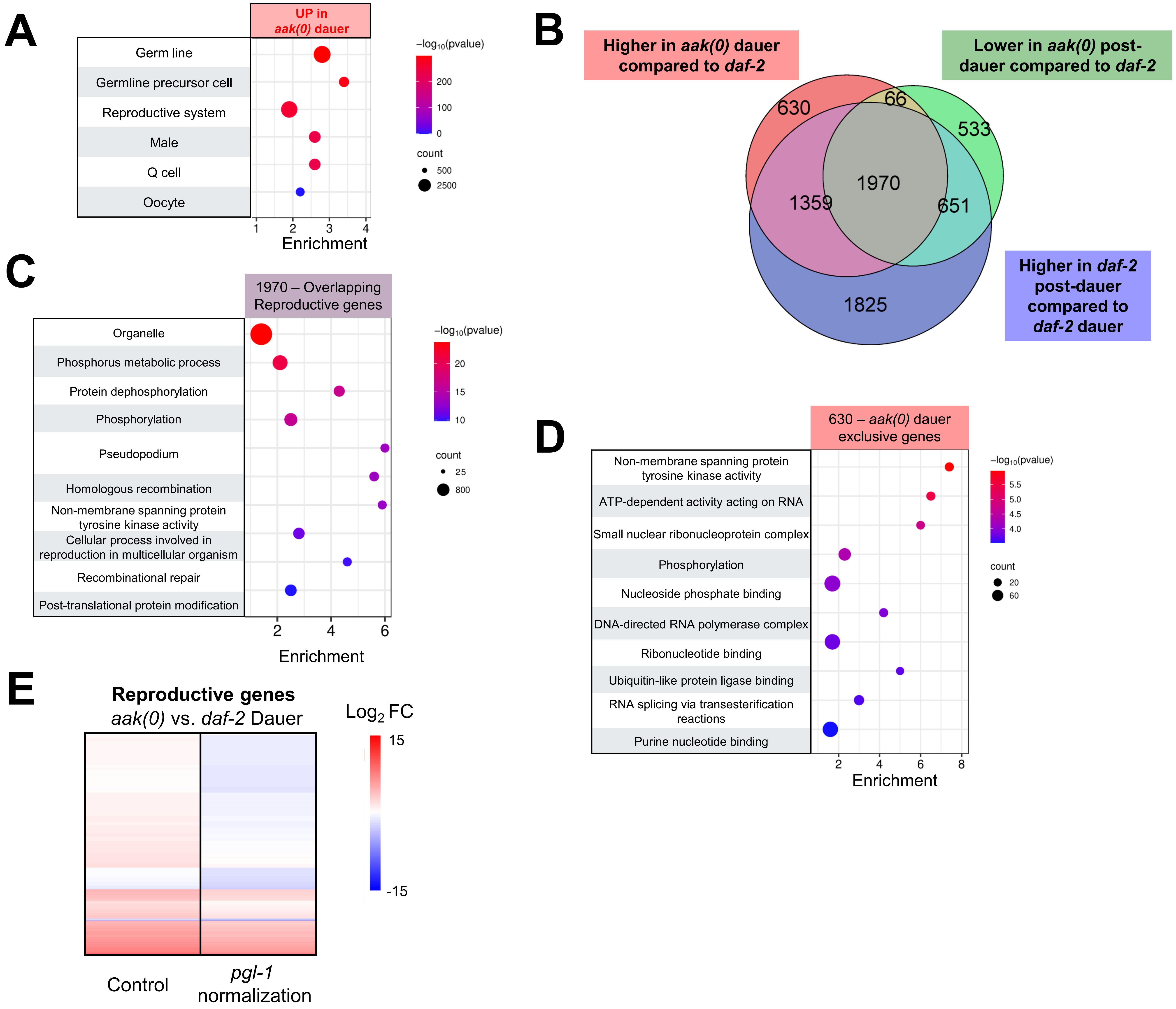

A
UP in
aak(0) dauer
Germ line
Germline precursor cell
Reproductive system
Male
Q cell
Oocyte
Enrichment
−log10(pvalue)
count
B
Higher in aak(0) dauer compared to daf-2
Lower in aak(0) post-dauer compared to daf-2
Higher in daf-2 post-dauer compared to daf-2 dauer
630
66
533
1970
1359
651
1825
C
1970 – Overlapping Reproductive genes
Organelle
Phosphorus metabolic process
Protein dephosphorylation
Phosphorylation
Pseudopodium
Homologous recombination
Non-membrane spanning protein tyrosine kinase activity
Cellular process involved in reproduction in multicellular organism
Recombinational repair
Post-translational protein modification
Enrichment
D
630 – aak(0) dauer exclusive genes
Non-membrane spanning protein tyrosine kinase activity
ATP-dependent activity acting on RNA
Small nuclear ribonucleoprotein complex
Phosphorylation
Nucleoside phosphate binding
DNA-directed RNA polymerase complex
Ribonucleotide binding
Ubiquitin-like protein ligase binding
RNA splicing via transesterification reactions
Purine nucleotide binding
Enrichment
E
Reproductive genes
aak(0) vs. daf-2 Dauer
Log2 FC
15
-15
Control
pgl-1 normalization

Figure S2. Widespread transcriptomic changes in *aak(0)* dauer and post-dauer animals largely affect reproductive genes

A) Bubble plots depicting tissue enrichment in *aak(0)* dauer larvae compared to *daf-2* controls, ranked by significance based on p-value. The size of bubbles indicates the number of genes in their respective categories that were enriched in the indicated dataset. Tissue enrichment was conducted using the Wormbase Gene Set Enrichment Analysis using q-value threshold of 0.1 (1,2).

B) Venn diagram of three gene expression datasets. Left: Increased expression in *aak(0)* dauer larvae compared to *daf-2* controls. Right: Decreased expression in *aak(0)* post-dauer animals compared to *daf-2* controls. Bottom: Increased expression in *daf-2* post-dauer animals compared to *daf-2* dauer. 1970 genes are found in all three subsets.

C) GO enrichment of the 1970 overlapping genes from the three-way Venn diagram. Terms ranked by significance based on p-value. Size of bubbles indicates the number of genes in their respective categories that were enriched in the indicated dataset. GO enrichment was conducted using the Wormbase Gene Set Enrichment Analysis using q-value threshold of 0.1 (1,2).

D) GO enrichment of the 630 *aak(0)* dauer specific enriched genes from three-way Venn diagram. Terms ranked by significance based on p-value. The size of the bubbles is proportional to the number of genes in their respective categories that were enriched in the indicated dataset. GO enrichment was conducted using the Wormbase Gene Set Enrichment Analysis using q-value threshold of 0.1 (1,2).

E) Heatmap depicting the expression of reproductive genes, highlighting differential expression between *aak(0)* dauer and post-dauer animals compared to *daf-2* controls with and without normalization to *pgl-1* values. Reproductive genes were selected from all differentially expressed genes in the *aak(0)* vs. *daf-2* comparisons for both dauer and post-dauer, and identified as ‘reproductive’ genes based on associated GO terms. Data represents Log_2 fold change in *aak(0)* vs. *daf-2* animals. Left heatmap depicts regular fold change, right depicts the new fold change after subtraction of *pgl-1* fold change.

All animals were assessed in a *daf-2* genetic background.

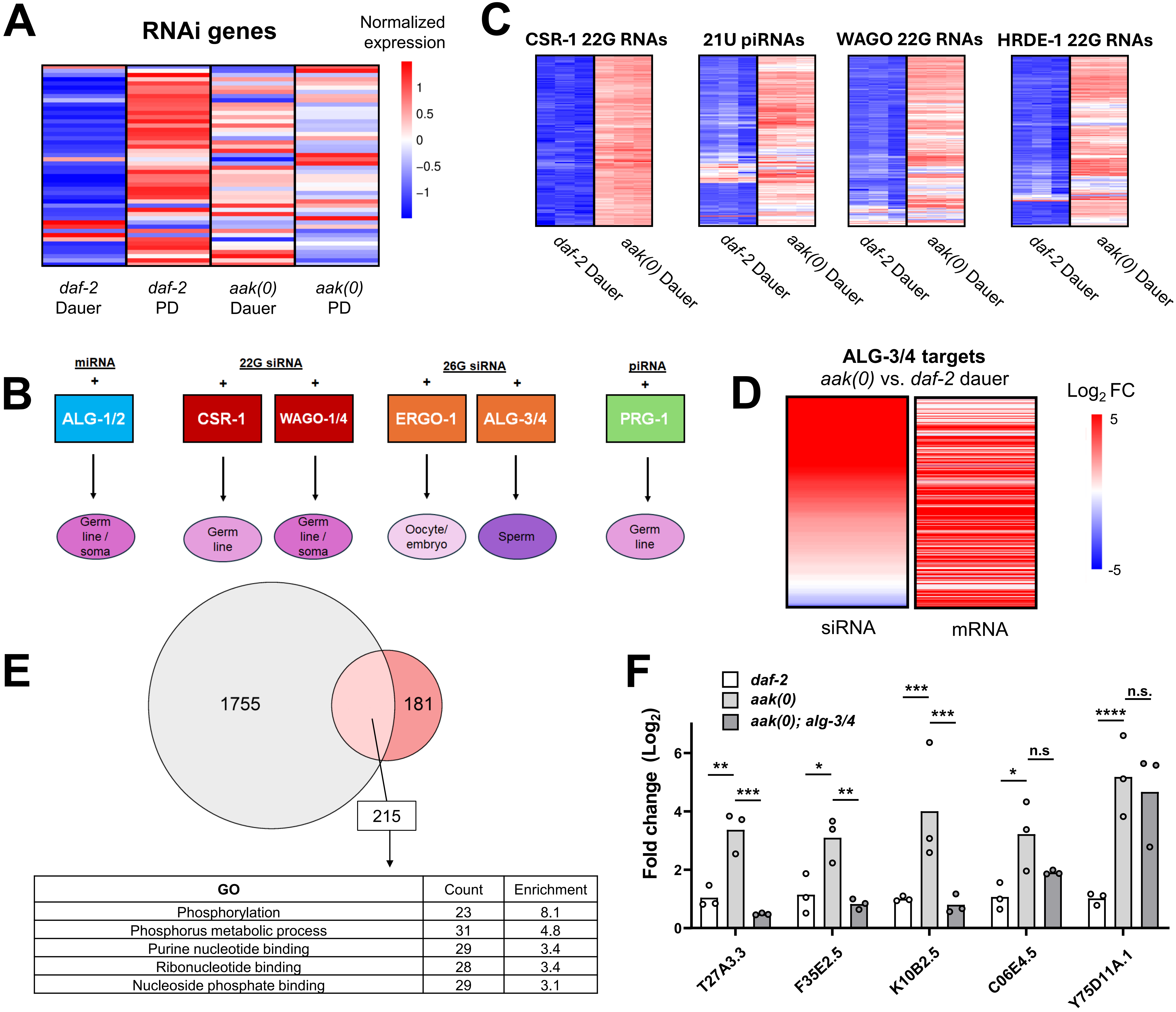

GO	Count	Enrichment
Phosphorylation	23	8.1
Phosphorus metabolic process	31	4.8
Purine nucleotide binding	29	3.4
Ribonucleotide binding	28	3.4
Nucleoside phosphate binding	29	3.1

Figure S3. RNAi pathway genes have elevated expression in AMPK dauer larvae

A) Heatmap of the expression of 51 RNAi pathway genes in *daf-2* and *aak(0)* dauer and post-dauer animals, generated by R. Genes listed in Supplementary Table 2 (3). Data represents normalized transcripts per million values for each gene, with higher relative expression in red and lower in blue.

B) Summary of major small RNA pathways in *C. elegans*. Different small RNA species bind to their respective Argonautes to influence gene expression in the indicated tissues. (4).

C) Heatmaps depicting normalized expression of small RNAs in *aak(0)* and *daf-2* dauer animals, separated by pathway. Data represents normalized transcripts per million values for each gene, with higher relative expression in red and lower in blue.

D) Heatmaps showing Log_2 fold change of ALG-3/4 26G siRNAs (left) and expression of their cognate gene targets (right) in *aak(0)* dauer animals. Values depict fold change in *aak(0)* vs. *daf-2* dauer animals.

E) Top: Venn diagram comparing 1970 AMPK-dependent reproductive genes from Fig S2B with a list of ALG-3/4 26G gene targets. Bottom: GO enrichment of 216 overlapping genes. 'Count' indicates number of genes out of 216 matching respective term. GO enrichment was conducted using the Wormbase Gene Set Enrichment Analysis using q-value threshold of 0.1

F) Expression levels of 26G siRNA levels in *aak(0)* and *aak(0); alg-3/4* dauer animals. Quantitative PCR was performed using TaqMan small RNA assays. U18 snoRNA was used as a control for ΔCt calculations, and all data were normalized to *daf-2* dauer values. Three replicates per group, ***$p < 0.001$ using one-way ANOVA for comparisons. Units are Log_2 fold change. See Supplementary Table 1 for primer sequences.

All animals were assessed in a *daf-2* genetic background.

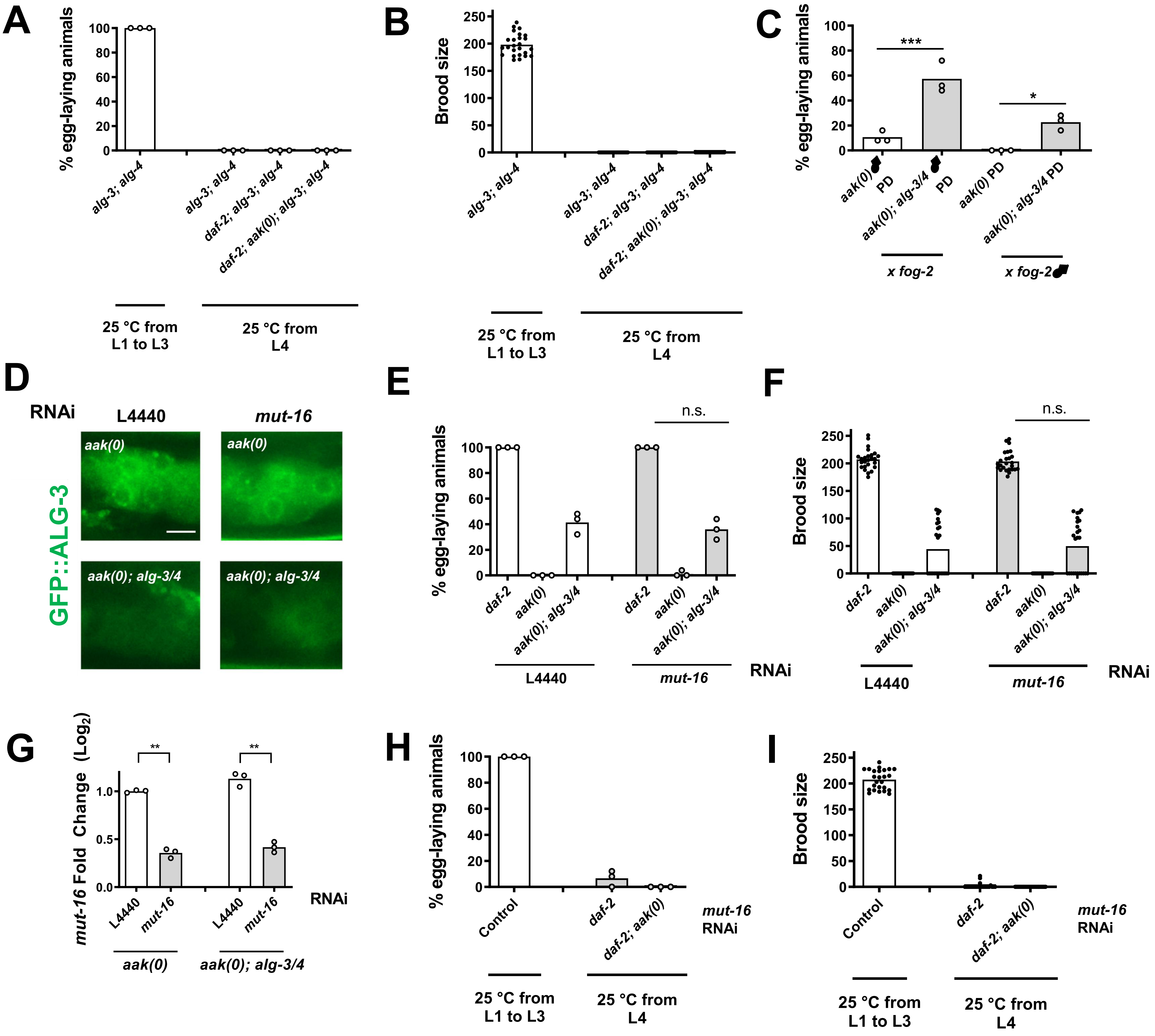
A
% egg-laying animals
100
80
60
40
20
0
alg-3; alg-4
alg-3; alg-4
daf-2; alg-3; alg-4
daf-2; aak(0); alg-3; alg-4
25 °C from L1 to L3
25 °C from L4
B
Brood size
250
200
150
100
50
0
alg-3; alg-4
alg-3; alg-4
daf-2; alg-3; alg-4
daf-2; aak(0); alg-3; alg-4
25 °C from L1 to L3
25 °C from L4
C
% egg-laying animals
***
*
aak(0) PD
aak(0); alg-3/4 PD
aak(0) PD
aak(0); alg-3/4 PD
x fog-2
x fog-2
D
RNAi
L4440
mut-16
aak(0)
aak(0)
aak(0); alg-3/4
aak(0); alg-3/4
GFP::ALG-3
E
% egg-laying animals
n.s.
daf-2
aak(0)
aak(0); alg-3/4
daf-2
aak(0)
aak(0); alg-3/4
L4440
mut-16
RNAi
F
Brood size
n.s.
daf-2
aak(0)
aak(0); alg-3/4
daf-2
aak(0)
aak(0); alg-3/4
L4440
mut-16
RNAi
G
mut-16 Fold Change (Log2)
**
**
1.0
0.5
0.0
L4440
mut-16
L4440
mut-16
RNAi
aak(0)
aak(0); alg-3/4
H
% egg-laying animals
Control
daf-2
daf-2; aak(0)
mut-16 RNAi
25 °C from L1 to L3
25 °C from L4
I
Brood size
Control
daf-2
daf-2; aak(0)
mut-16 RNAi
25 °C from L1 to L3
25 °C from L4

Figure S4. Loss of ALG-3/4 in *aak(0)* animals partially restores male fertility, and the effects on *aak(0)* animals are not dependent on *mut-16*

A-B) Post-dauer fertility and brood size of *alg-3; alg-4* mutants. Bleach-synchronized animals were either grown at 25 °C from L1 to L3 before being shifted to 15 °C for reproductive development; or were grown at 15 °C and shifted to 25 °C for the L4 stage before being returned to 15 °C for reproductive development. For brood size assays, animals were singled and brood size of each individual animal was measured.

C) Fertility of *aak(0)* or *aak(0); alg-3/4* post-dauer animals crossed with *fog-2.* White: Post-dauer males from indicated strains were crossed with *fog-2* females. Grey: Post-dauer hermaphrodites from indicated strains were crossed with *fog-2* males. When setting up crosses, post-dauer animals were staged by phenotype rather than time. A ratio of 5 males to 1 hermaphrodite/female was used per plate. The presence of male progeny was assessed after several days to determine percentage of egg-laying animals that mated. n=25 hermaphrodites/females for each trial. *$p < 0.05$, ***$p < 0.001$ using one-way ANOVA for the indicated comparisons.

D) Representative confocal micrographs of GFP::ALG-3 expression in the germ line of *aak(0)* (Top) and *aak(0); alg-3/4* (Bottom) dauer larvae, subjected to *mut-16* RNAi or L4440 empty vector control. Synchronized L1 animals were put onto RNAi plates at 25 °C for 96 hours before being imaged. All animals are in the *daf-2* background. Scale bar = 10 µm.

E-F) Post-dauer fertility and brood size of *aak(0)* or *aak(0); alg-3/4* mutants following RNAi against *mut-16* or L4440 empty vector control. For statistical comparisons, *mut-16* RNAi treated samples were compared to their respective control (L4440) samples.

Animals were grown at 25 °C from synchronized L1s for 96 hours before being singled onto plates. After approximately a week, fertility of each animal was assessed and the total % of egg-laying animals per sample was recorded. For brood size assays, animals were singled and brood size of each individual animal was measured.

G) Gene expression of *mut-16* in *aak(0)* and *aak(0); alg-3/4* dauer animals treated with RNAi against *mut-16* or the L4440 empty vector as determined by RT-qPCR. Primers against *mut-16* were used, while *tba-2* was used as a housekeeping gene for comparison. Total RNA was collected after 48 hours in dauer. All animals were maintained in a *daf-2* genetic background. Data from *mut-16* samples were compared to L4440 control samples for statistical tests. 3 independent trials were performed for all gene expression assays. **$p < 0.001$ using two-way ANOVA.

H-I) Post-dauer fertility and brood size of *aak(0)* or *aak(0)* mutants following RNAi against *mut-16* or L4440 empty vector control. Bleach-synchronized animals were either grown at 25°C from L1 to L3 before being shifted to 15°C for reproductive development; or were grown at 15 °C and shifted to 25 °C for the L4 stage before being returned to 15 °C for reproductive development. For brood size assays, animals were singled and brood size of each individual animal was measured.

Post-dauer fertility data represent three independent trials, with the mean represented by columns and values for individual trials indicated by small circles. n=50 for each trial of post-dauer fertility, n=25 for brood size assays.

All animals in D) to I) were assessed in a *daf-2* genetic background.

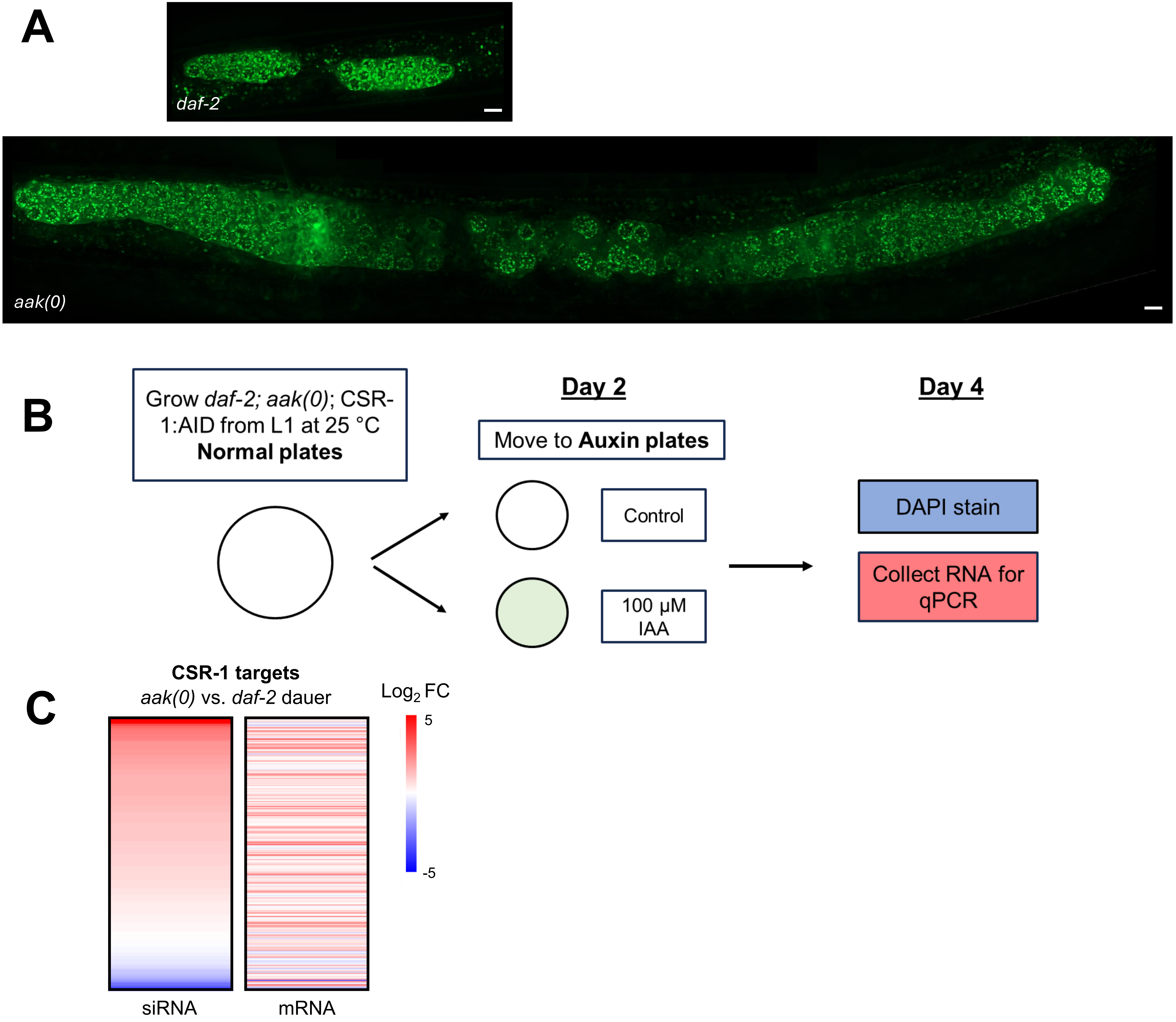

A
daf-2
aak(0)
B
Grow daf-2; aak(0); CSR-1:AID from L1 at 25 °C
Normal plates
Day 2
Move to Auxin plates
Control
100 µM IAA
Day 4
DAPI stain
Collect RNA for qPCR
C
CSR-1 targets
aak(0) vs. daf-2 dauer
Log2 FC
5
-5
siRNA
mRNA

Figure S5. CSR-1 is expressed strongly in the germline of *daf-2* and *aak(0)* dauer larvae

A) Representative confocal micrographs depicting CSR-1::GFP expression in the germ lines of dauer larvae in *daf-2* (Top) or *aak(0)* (Bottom) animals after 96 hours of growth. Scale bar = 10 μm.

B) Protocol for Auxin treatment of CSR-1::AID strains. L1 synchronized animals with the CSR-1::AID tag were grown at 25 °C on NGM plates for 46 hours before half were transferred to a fresh set of normal NGM plates, and the other half transferred to NGM plates supplemented with 100 μM IAA. After 48 hours on these plates, animals were collected for DAPI staining or RNA extraction.

C) Heatmaps showing Log_2 fold change of CSR-1 22G siRNAs (left) and expression of their cognate gene targets (right) in *aak(0)* dauer animals. Values depict fold change in *aak(0)* vs. *daf-2* dauer animals.

All animals were assessed in a *daf-2* genetic background.

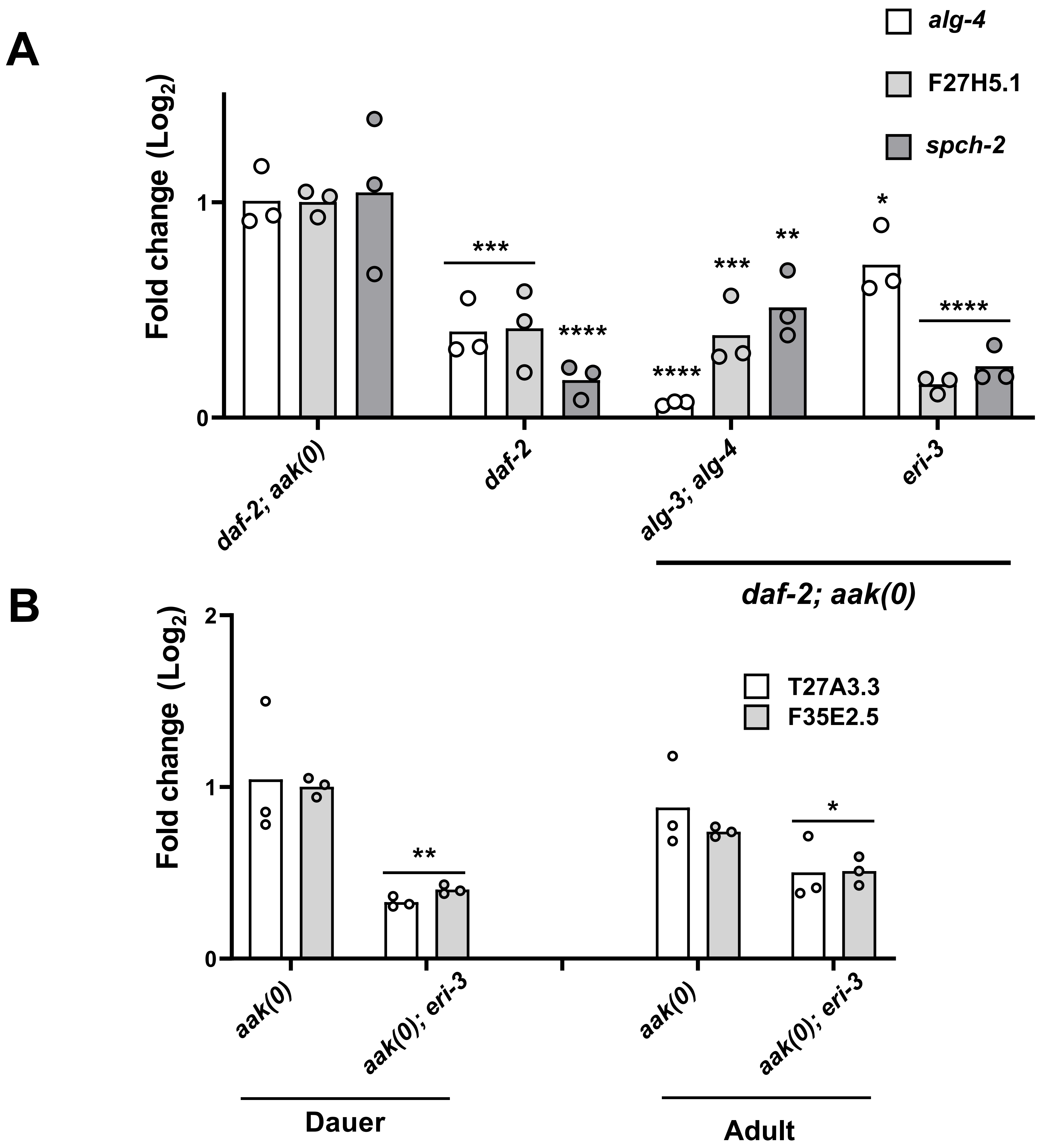
A
alg-4
F27H5.1
spch-2
Fold change (Log2)
1
0
***
****
****
***
**
*
****
daf-2; aak(0)
daf-2
alg-3; alg-4
eri-3
daf-2; aak(0)
B
Fold change (Log2)
2
1
0
T27A3.3
F35E2.5
**
*
aak(0)
aak(0); eri-3
aak(0)
aak(0); eri-3
Dauer
Adult

Figure S6. Loss of *eri-3* corrects gene expression and reduces 26G small RNA levels in *aak(0)* animals

A) Expression levels of *alg-4,* F27H5.1 and *spch-2* in the dauer stage of *daf-2, daf-2; aak(0), daf-2; aak(0); alg-3/4* and *daf-2; aak(0); eri-3* mutants. L1 synchronized animals were grown at 25 °C for 96 hours before being collected for RNA extraction. RT-qPCR was performed using primers against indicated genes. *tba-2* was used as a housekeeping gene for ΔCt calculations, and all data were normalized to *daf-2; aak(0)* values. Statistical comparisons were done against equivalent *daf-2; aak(0)* data for each gene. Three replicates per group, ****p < 0.0001, ***p < 0.001, **p < 0.01, *p < 0.05 using one-way ANOVA for comparisons. Units are Log_2 fold change.

B) Expression levels of T27A3.3 and F35E2.5 siRNA levels in *daf-2; aak(0)* and *daf-2; aak(0); eri-3* dauer and adult animals. For dauer, L1 synchronized animals were grown at 25 °C for 96 hours before being collected for RNA extraction, whereas adults were grown at 15 °C for 96 hours. Quantitative PCR was performed using TaqMan small RNA assays. U18 snoRNA was used as a control for ΔCt calculations, and all data were normalized to *aak(0)* dauer values. Statistical comparisons were performed in *aak(0); eri-3* samples using equivalent *aak(0)* data for each siRNA. Three replicates per group, ****p < 0.0001, ***p < 0.001, **p < 0.01 *p < 0.05 using one-way ANOVA for comparisons. Units are Log_2 fold change.

All animals were assessed in a *daf-2* genetic background.

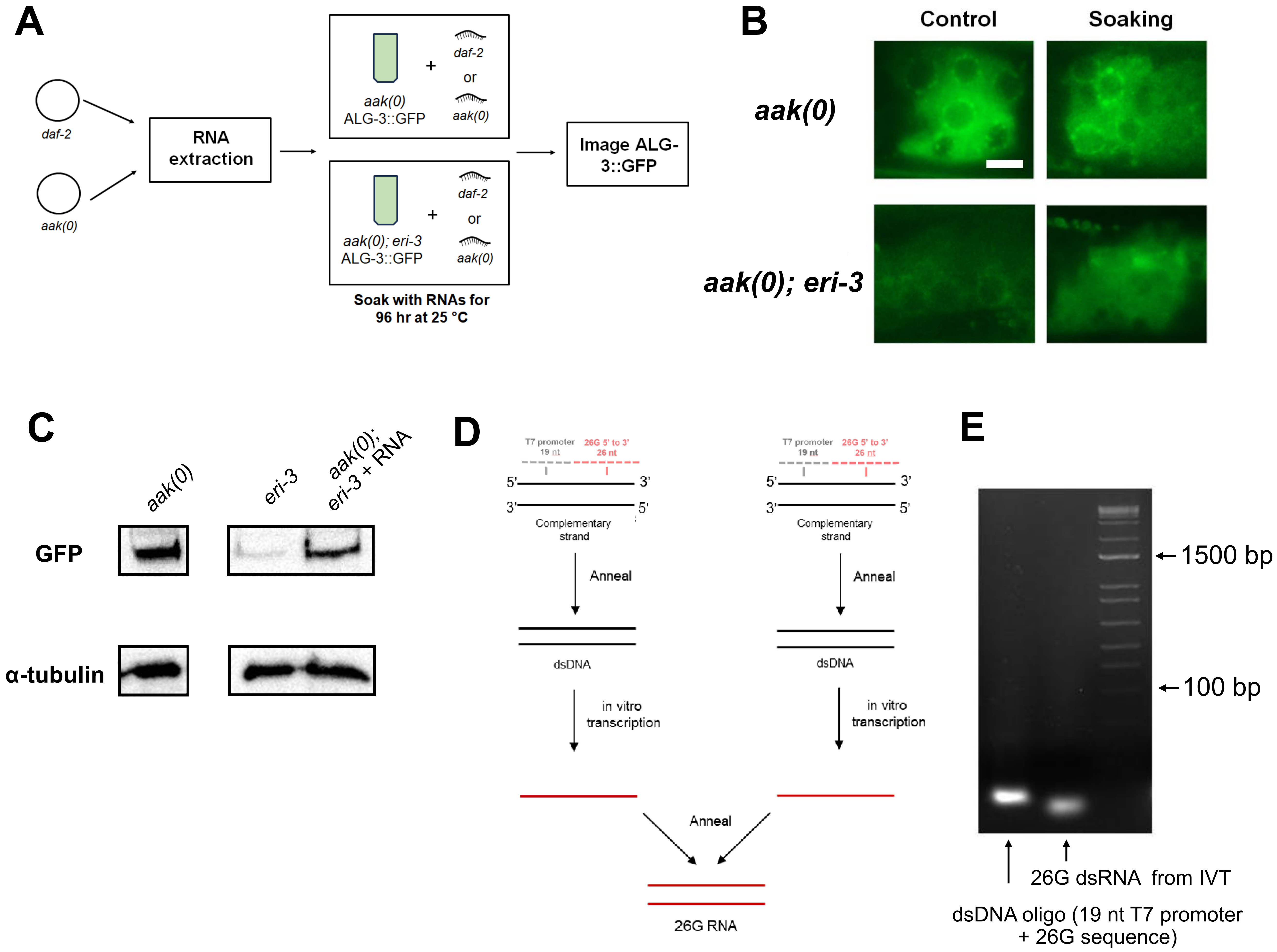
A
daf-2
aak(0)
RNA extraction
aak(0) ALG-3::GFP
+
daf-2
or
aak(0)
aak(0); eri-3 ALG-3::GFP
Soak with RNAs for 96 hr at 25 °C
Image ALG-3::GFP
B
Control
Soaking
aak(0)
aak(0); eri-3
C
aak(0)
eri-3
aak(0); eri-3 + RNA
GFP
α-tubulin
D
T7 promoter 19 nt
26G 5' to 3' 26 nt
5'
3'
Complementary strand
Anneal
dsDNA
in vitro transcription
26G RNA
E
1500 bp
100 bp
26G dsRNA from IVT
dsDNA oligo (19 nt T7 promoter + 26G sequence)

Figure S7. Soaking *aak(0); eri-3* animals with RNAs alters GFP::ALG-3 levels

A) Protocol for soaking of animals with total RNA extracted from *daf-2* or *aak(0)* animals, adapted from Wong et al, 2024 (5). RNA was extracted from *daf-2* or *aak(0)* dauer populations. *aak(0)* or *aak(0); eri-3* animals were synchronized in L1, then grown in liquid culture with M9 and HB101 bacteria along with RNA extracted from either *daf-2* or *aak(0)* dauers. After 96 hours of growth at 25 °C, animals were harvested for either confocal imaging or Western blot.

B) Representative confocal micrograph images depicting GFP::ALG-3 expression in *aak(0)* (Top) or *aak(0); eri-3* (Bottom) dauer larvae following soaking. Larvae were grown in liquid from eggs and treated with RNA extracted from either *daf-2* animals (Left) or *aak(0)* animals (Right) for 96 hours at 25 °C. Animals were put onto plates briefly before being picked into levamisole on agarose pads on glass slides for imaging. Scale bar = 10 µm.

C) Representative Western blot measuring levels of GFP::ALG-3. Left: *aak(0); eri-3* dauer larvae with and without soaking with RNA extracted from *aak(0)* dauers after 96 hours growth in liquid at 25 °C. Right: *aak(0)* and *aak(0); eri-3* dauer larvae. All animals were in a *daf-2* background. Antibodies against GFP were used, with antibodies against α-tubulin as a loading control.

D) Protocol for the generation of 26G RNAs in vitro, adapted from Zhang et al, 2004 (6). Double-stranded DNA oligonucleotides were generated, containing the T7 RNA Polymerase sequence along with the desired 26G RNA sequence in either a Sense or Antisense sequence. Separate in vitro transcription reactions were carried out to generate the Sense and Antisense RNA strand, which were then annealed to create a single 26G RNA.

E) Gel electrophoresis blot showing a 45 nt long band of double-stranded DNA oligonucleotide used as a template for *in vitro* synthesis (Left), an annealed double-stranded 26G RNA generated through in vitro transcription (Center), and a 1 Kb plus DNA ladder (Right).

All animals were assessed in a *daf-2* genetic background.

	Primer name	Sequence	Primer name	Sequence	
**RT-qPCR**					
tba-2	*tba-2* q 5’	TCACCAATAGCTGCTTCGAG	*tba-2* q 3’	ACGTCCTTTGGAACGACG	
alg-4	*alg-4* q 5’	TCGGAGTTGCTGTGTCTTCT	*alg-4* q 3’	CAAGTGGCAGGCATGTACAG	
spch-2	*spch-2* q 5’	CTCAGTCCGCTCGATCTCAA	*spch-2* q 3’	AGACGCTTCTCACAACCGA	
ssp-16	*ssp-16* q 5’	ATCTGGATCCAAGGACGTCG	*ssp-16* q 3’	CTGGTGCGAAATGAACGACA	
egg-2	*egg-2* q 5’	CTCAGGAAGCGATGCATGTC	*egg-2* q 3’	AGACTTTGCTGGCTTTTATGC	
gpdh-2	*gpdh-2* q 5’	TCCACATCAATTCGTCAAAGGA	*gpdh-2* q 3’	TTCAGTCCTCCACGCTTTTC	
F27H5.1	F27H5.1 q 5’	AAGTCCTCCAAGAAGTGCGA	F27H5.1 q 3’	TCCGGGAATGTGTACTTCGA	
mut-16	*mut-16* q 5’	GGCGCATTCTTTCCAGTCATT	*mut-16* q 3’	CGGATTCAAATGTATCTGCTTCC	
**Taqman**					
T27A3.3	26G-S5 T27A3.3	GTGGATCAGCAGTGAGCGACATGGTA	K10B2.5	26G-SR2 K10B2.5	GATAGAAGGGTACATAAAATTCTAAT
F35E2.5	26G-S8 F35E2.5	GAAAATGAAGTAGAATATGATCATCG	Y75D11A.1	26G-SR3 Y75D11A.1	GTAGAAAGAGAACATAGTAGAAGTCT
C06E4.5	26G-SR1 C06E4.5	GTGAAATTTCGAGCGTATAGCACCAA			
**in vitro 26G**					
ssp-16	*ssp-16* IVT Sense 5’	GGTAATACGACTCACTATAGTGGATCAGCAGTGAGCGACATGGTA	*ssp-16* IVT Sense 3’	TACCATGTCGCTCACTGCTGATCCACTATAGTGAGTCGTATTACC	
	ssp-16 IVT Antisense 5’	GGTAATACGACTCACTATATACCATGTCGCTCACTGCTGATCCAC	*ssp-16* IVT Antisense 3’	GTGGATCAGCAGTGAGCGACATGGTATATAGTGAGTCGTATTACC	
spch-2	*spch-2* IVT Sense 5’	GGTAATACGACTCACTATAGTTCTTCTGACTCTTCGACTTCGGCA	*spch-2* IVT Sense 3’	TGCCGAAGTCGAAGAGTCAGAAGAACTATAGTGAGTCGTATTACC	
	spch-2 IVT Antisense 5’	GGTAATACGACTCACTATATGCCGAAGTCGAAGAGTCAGAAGAAC	*spch-2* IVT Antisense 3’	GTTCTTCTGACTCTTCGACTTCGGCATATAGTGAGTCGTATTACC	
msrp-2	*msrp-2* IVT Sense 5’	GGTAATACGACTCACTATAGAGAACAACTGTGGAAATGGCTCCAA	*msrp-2* IVT Sense 3’	TTGGAGCCATTTCCACAGTTGTTCTCTATAGTGAGTCGTATTACC	
	msrp-2 IVT Antisense 5’	GGTAATACGACTCACTATATTGGAGCCATTTCCACAGTTGTTCTC	*msrp-2* IVT Antisense 3’	GAGAACAACTGTGGAAATGGCTCCAATATAGTGAGTCGTATTACC	

Supplementary Table 1 – List of primers

List of all primers and their sequences that were used in the study for RT-qPCR, Taqman, and *in vitro* 26G RNA synthesis.

RNAi pathway	Protein	RNAi pathway	Protein
ERGO-1 class 26G RNA	ERGO-1	NRDE-3-associated 22G RNA	NRDE-1
	HENN-1		NRDE-2
	ERI-9		NRDE-4
	ERI-6	HRDE-1-associated 22G RNA	HRDE-1
	ERI-7		HPL-2
	MUT-16		MES-4
ALG-3/4 class 26G RNA	ALG-3		SET-32
	ALG-4		SET-25
WAGO 22G RNA	WAGO-1	Biogenesis factors	DRH-3
	WAGO-4		ERI-5
	MUT-16		DCR-1
	MUT-7		ERI-1
	MUT-2		ERI-3
	MUT-15		RDE-4
	MUT-14		RRF-1
	RDE-10		EGO-1
	RDE-11		FKH-3
CSR-1 22G RNA	CSR-1		FKH-4
	EKL-1		FKH-5
21U RNA (Type I)	PRG-1		DRSH-1
miRNAs	ALG-1		PASH-1
	ALG-2		
	AIN-1		
	AIN-2		
	NHL-2		
	CGH-1		
	TSN-1		
	VIG-1		
	XRN-2		
	DCS-1		

Supplementary Table 2 – RNA interference pathway components

A list of RNAi pathway genes that were analyzed in the transcriptomic data set to identify changes in expression level in dauer vs. post-dauer of *daf-2* and *aak(0)* animals (3).

acs-4	*ect-2*	*mcm-7*	***rme-2***
air-1	*efa-6*	***mei-2***	***rnp-8***
akir-1	***egg-6***	*mel-28*	*rnr-1*
ani-2	*pold-1*	***mes-4***	***rod-1***
edg-1	*rif-1.1*	***mex-1***	*rpn-2*
gex-5	*ctf-4*	***mex-5***	*smc-3*
**C01G8.1**	**F45F2.11**	***mex-6***	*smc-4*
simr-1	**F46B6.5**	***mom-2***	***smc-6***
**C36B1.11**	F49C12.9	***mre-11***	***spd-2***
cbd-1	**F54D11.4**	***mrp-7***	***spn-4***
cdc-25.1	*spt-16*	***msh-6***	*szy-20*
cdl-1	**F55D12.5**	*nmy-2*	T05F1.2
ced-3	F59E12.1	***oma-1***	T07F8.4
cee-1	*gfat-1*	*patr-1*	**T13F2.6**
cgh-1	***gld-1***	***perm-1***	T19B4.5
cpb-1	***gld-3***	***pgl-1***	**T24D1.3**
cpb-3	***glp-1***	***plk-3***	***ntl-2.2***
cpg-1	*hcp-1*	***parp-1***	*tpxl-1*
cyb-1	*him-1*	***prg-1***	*usp-48*
cye-1	***hpo-40***	***ptr-2***	***wago-1***
cyk-4	***ify-1***	***puf-3***	*wee-1.3*
cyk-7	***ima-2***	***puf-5***	***xnd-1***
**D1044.6**	*isw-1*	***puf-6***	***xpc-1***
lotr-1	*knl-1*	***puf-7***	**Y57A10A.31**
daf-18	*lex-1*	*ran-3*	**Y65A5A.2**
daz-1	*lin-53*	*rcor-1*	**Y82E9BR.19**
deps-1	*mcm-3*	*rde-4*	*zyg-9*
dpl-1	*mcm-6*	***rga-3***	***chs-1***

Supplementary Table 3 – Many 'Seesaw' genes affected by CSR-1 compromise also overlap with AMPK-dependent reproductive genes

A list of "Seesaw" genes defined in the 'WTPhe up::WTStv down' subset from Ow et al, 2018 (7) that overlap with ~2000 AMPK-dependent genes that dynamically change expression in aak(0) mutant dauer and post-dauer. Genes associated with precursor germline cells, based on tissue enrichment analysis, highlighted in grey.

SUPPLEMENTARY REFERENCES

1. Angeles-Albores,D., Raymond, Chan,J. and Sternberg,P.W. (2016) Tissue enrichment analysis for *C. elegans* genomics. BMC Bioinformatics, 17.

2. Angeles-Albores,D., Raymond Y.W. Lee, Chan,J. and Sternberg,P.W. (2018) Two new functions in the WormBase Enrichment Suite. MicroPubl Biol, 17912/W25Q2N.

3. Billi,A.C., Fischer,S.E. and Kim,J.K. (2010) Endogenous RNAi pathways in *C. elegans.* WormBook, 1-49.

4. Hoogstrate,S.W., Volkers,R.J., Sterken,M.G., Kammenga,J.E. and L Basten Snoek (2014) Nematode endogenous small RNA pathways. Worm, 3, e28234–e28234.

5. Wong,C., Jurczak,E.M. and Roy,R. (2024) Neuronal exosomes transport an miRISC cargo to preserve stem cell integrity during energy stress. Cell Rep., 43, 114851–114851.

6. Zhang,S.-Q. (2004) Polymerase synthesis and potential interference of a small-interfering RNA targeting hPim-2. World J. Gastroenterol., 10, 2657.

7. Ow,M.C., Borziak,K., Nichitean,A.M., Dorus,S. and Hall,S.E. (2018) Early experiences mediate distinct adult gene expression and reproductive programs in *Caenorhabditis elegans*. PLOS Genet., 14, e1007219.
